# In vivo identification of *Drosophila* rhodopsin interaction partners by biotin proximity labeling

**DOI:** 10.1038/s41598-024-52041-3

**Published:** 2024-01-23

**Authors:** Nilofar Feizy, Sarah Franziska Leuchtenberg, Christine Steiner, Berit Würtz, Leo Fliegner, Armin Huber

**Affiliations:** 1 Department of Biochemistry, Institute of Biology, University of Hohenheim, Stuttgart, Germany; 2 Mass Spectrometry Unit, Core Facility Hohenheim, University of Hohenheim, Stuttgart, Germany

**Keywords:** G protein-coupled receptors, Proteome, Proteins, Proteomics, Protein-protein interaction networks, Membrane trafficking, Protein transport, Visual system, Molecular neuroscience

## Abstract

Proteins exert their function through protein–protein interactions. In *Drosophila*, G protein-coupled receptors like rhodopsin (Rh1) interact with a G protein to activate visual signal transduction and with arrestins to terminate activation. Also, membrane proteins like Rh1 engage in protein–protein interactions during folding within the endoplasmic reticulum, during their vesicular transport and upon removal from the cell surface and degradation. Here, we expressed a Rh1-TurboID fusion protein (Rh1::TbID) in *Drosophila* photoreceptors to identify in vivo Rh1 interaction partners by biotin proximity labeling. We show that Rh1::TbID forms a functional rhodopsin that mediates biotinylation of arrestin 2 in conditions where arrestin 2 interacts with rhodopsin. We also observed biotinylation of Rh1::TbID and native Rh1 as well as of most visual signal transduction proteins. These findings indicate that the signaling components in the rhabdomere approach rhodopsin closely, within a range of ca. 10 nm. Furthermore, we have detected proteins engaged in the maturation of rhodopsin and elements responsible for the trafficking of membrane proteins, resembling potential interaction partners of Rh1. Among these are chaperons of the endoplasmic reticulum, proteins involved in Clathrin-mediated endocytosis as well as previously unnoticed contributors to rhodopsin transportation, such as Rab32, Vap33, or PIP82.

## Introduction

Rhodopsins are prototypical G protein-coupled receptors that initiate visual signal transduction cascades. In fly photoreceptors the major rhodopsin Rh1 is expressed in R1–6 photoreceptor cells^1,2^. Light-activation of Rh1 converts rhodopsin into its active state, termed metarhodopsin, and triggers a signaling cascade mediated by a Gq protein (composed of a Gαq and a Gβγ subunit) and phospholipase Cβ (PLCβ) that results in the opening of the transient receptor potential channels TRP and TRPL (for review see^3–5^). Inactivation of metarhodopsin is achieved by binding of two arrestin variants: arrestin 1 and arrestin 2. Arrestin 2 is much more abundant than arrestin 1 and contributes the most to metarhodopsin inactivation^6–9^. Arrestins bind to the cytosolic region of metarhodopsin and thereby inhibit further activation of the visual Gq protein. Accordingly, during visual transduction, direct interaction partners of Rh1 rhodopsin include the visual Gq protein, arrestin 1 and arrestin 2.

Besides these interaction partners in signal transduction, Rh1 binds to proteins involved in protein maturation and transport. Rh1 is synthesized into the membrane of the rough endoplasmic reticulum (ER) where it interacts with ER chaperones such as calnexin 99A, NinaA, and Xport-A and -B (for review see^10^). Next, it is transported via the Golgi apparatus to the base of the rhabdomere, a stack of densely packed microvilli at the apical side of photoreceptor cells, which forms the light sensitive compartment. Activated Rh1 is endocytosed and mostly degraded in the lysosome except for a fraction that becomes recycled and returns to the rhabdomere via a retromer-dependent pathway^11,12^. Numerous interaction partners are assumed to be involved in these Rh1 maturation and transport processes. Failures in rhodopsin inactivation or in rhodopsin transport have detrimental effects and result in photoreceptor degeneration. For example, arrestin 2 null mutants cannot inactivate the visual transduction cascade resulting in continuous Ca^2+^ influx through TRP channels and cell degeneration^6^. Accumulation of Rh1 either during anterograde or retrograde rhodopsin transport, mostly due to rhodopsin mutations affecting correct protein folding or defects in rhodopsin chaperones or transport proteins, results in toxic rhodopsin and rhodopsin-arrestin complexes also leading to cell degeneration^13,14^. In this context, *Drosophila* photoreceptors provide a precious tool to be used as a model system for human eye diseases such as Retinitis Pigmentosa, and more generally for neurodegenerative diseases including Alzheimer´s and Parkinson’s disease^13,15^.

In the present study we established a biotin proximity labeling system in *Drosophila* photoreceptors and employed it to identify in vivo interaction partners of the major rhodopsin Rh1. Biotin ligases biotinylate proteins in vivo which are in close proximity to and engage in constant or transient interactions with the bait protein^16,17^. We attached a biotin ligase, termed TurboID, to the C-terminus of Rh1 and expressed this fusion protein (Rh1::TbID) in *Drosophila* R1–6 photoreceptor cells. TurboID is an improved variant of the *Escherichia coli* biotin ligase BirA that adenylates biotin to form the reactive intermediate biotin–adenosine monophosphate (Biotin-5ʹ-AMP). Biotin-5ʹ-AMP diffuses from the active site of the biotin ligase and reacts with proteins within a radius of about 10 nm at a time scale of minutes^17^. We found that several components of the visual signal transduction cascade, including the ion channels TRP and TRPL, the visual Gq protein, and visual arrestins, as well as proteins required for rhabdomere structures became biotinylated in Rh1::TbID flies. In addition, we identified several proteins involved in rhodopsin maturation and components for membrane protein trafficking as putative Rh1 interaction partners. The latter include so far unrecognized players in rhodopsin transport like Rab32, Vap33, and the stalk-membrane protein PIP82.

## Results and discussion

### Generation of Rh1::TbID expressing transgenic *Drosophila*

**Figure 1:**
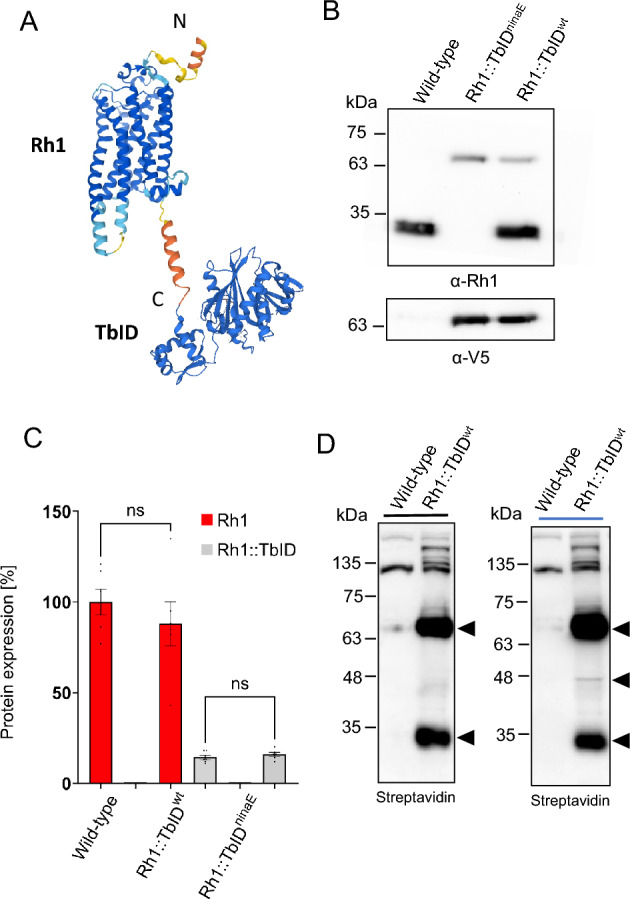
Generation of Rh1::TbID expressing transgenic *Drosophila.* (**A**) Scheme of a C-terminal Rh1::TbID fusion protein. The DNA encoding TurboID-V5 (TbID) was cloned 3ʹ of the coding region of Rh1 cDNA. AlphaFold structure prediction for *Drosophila* Rh1 and *E. coli* BirA was used to obtain the depicted models. N- and C-terminal regions of Rh1 are indicated. (**B**) Immunoblot analysis assessing expression of fusion protein Rh1::TbID in heads of transgenic flies. Rh1 and Rh1::TbID were detected by a polyclonal Rh1-antibody (α-Rh1). Rh1-TurboID expression was also detected by an α-V5 antibody. (**C**) Quantification of native Rh1 and Rh1::TbID in wild-type flies vs. flies expressing Rh1::TbID in wild-type background (Rh1::TbID^wt^) and in *ninaE*^*17*^ background (Rh1::TbID^ninaE^). Immunoblot analysis was carried out using α-Rh1. Rh1 level in wild type was set to 100% (n = 6, p > 0.05 = ns). (**D**) Western blot analysis of biotinylated proteins in wild-type heads and heads of transgenic flies (Rh1::TbID^wt^) that were either kept in the dark or illuminated with blue light for 4 h. Blots were probed with HRP-conjugated streptavidin. Transgenic flies revealed increased biotinylation of proteins compared to wild-type controls. Bands corresponding to the self-biotinylated Rh1::TbID, native Rh1, and the 48 kDa band present in blue light illuminated flies are marked with arrowheads. In all blots shown protein extracts from heads (equivalent of 2.4 heads per lane) were blotted after electrophoretic separation in 12% SDS gels. The size of molecular weight markers in kDa is indicated on the left. Original full size blots are presented in Supplementary Fig. 4S.

For identification of in vivo interaction partners of rhodopsin 1 (Rh1) via proximity labeling with the TurboID system, we created a DNA construct to express Rh1 fused with TbID-V5 in *Drosophila* photoreceptor cells R1–6 under control of the rhodopsin 1 promoter (Fig. 1A). We generated transgenic fly lines expressing Rh1::TbID in wild-type background containing both Rh1::TbID and native Rh1 (Rh1::TbID^wt^) as well as flies expressing Rh1::TbID in *ninaE*^*17*^ background containing no native Rh1 (Rh1::TbID^ninaE^). In order to verify expression of Rh1::TbID in photoreceptor cells, immunoblots were performed on protein extracts of fly heads. We used a polyclonal Rh1-antibody to detect Rh1 and Rh1::TbID, as well as an anti-V5 antibody to detect Rh1::TbID via its V5 tag (Fig. 1B). Rh1::TbID was detected at the expected molecular weight of ca. 65 kDa. A quantification of the immunoblot signals revealed lower expression of Rh1::TbID as compared to native Rh1 (Fig. 1C). The expression level of Rh1::TbID was 16% relative to the amount of native Rh1 found in transgenic flies. A similar amount of Rh1::TbID was detected in flies irrespective of the presence of native Rh1. To assess TbID functionality, we compared protein biotinylation in age-synchronized young adult flies expressing the *Rh1::TbID* transgene with wild-type controls (Fig. 1D). Flies were either kept in the dark or illuminated with blue light for 4 h to generate a maximum amount of metarhodopsin. In head extracts obtained from wild-type flies a prominent biotinylated protein at 110 kDa and several minor protein bands were observed, presumably representing native biotinylated proteins such as carboxylases or histones. In Rh1::TbID expressing flies additional prominent biotinylated protein bands were detected above the 110 kDa protein as well as at 65 kDa (presumably representing self-biotinylated Rh1::TbID), and at 32 kDa (representing mainly native Rh1, but see below). In addition, a biotinylated protein only present in blue light illuminated flies at 48 kDa was detected, which was later identified as arrestin 2.

### Rh1::TbID forms a functional rhodopsin

**Figure 2:**
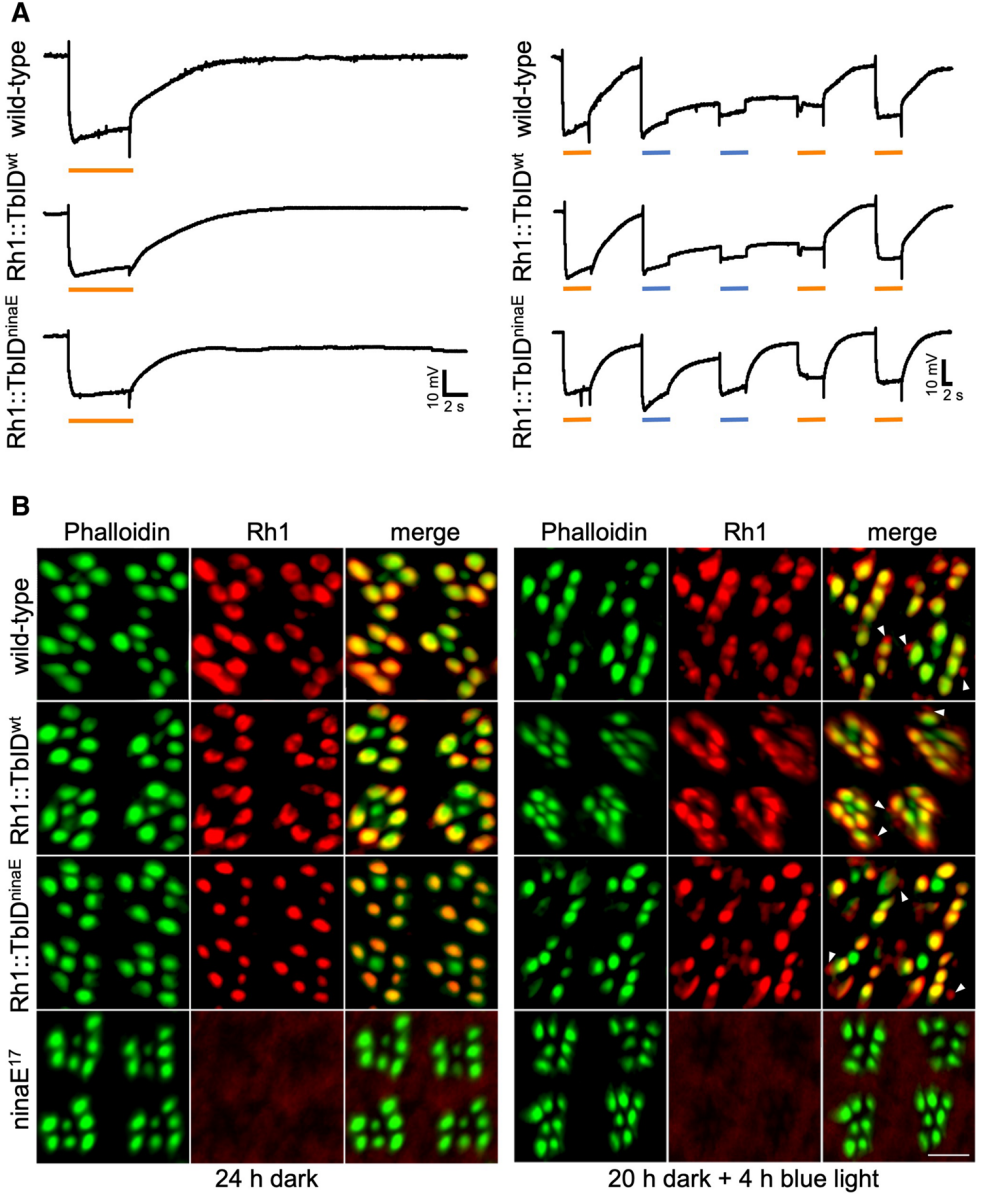
Rh1::TbID forms a functional rhodopsin in the rhabdomere. (**A**) Electroretinograms (ERG) of 3 days old wild-type and transgenic (Rh1::TbID^wt^ and Rh1::TbID^ninaE^) flies. Dark-adapted flies were subjected to a single orange light stimulus (left side) or to an OBBOO-protocol (right side, see “Materials and methods” section). Orange or blue light stimuli are indicated by orange and blue bars, respectively. Flies containing Rh1::TbID and no native Rh1 (Rh1::TbID^ninaE^) show wild-type light responses except for a less pronounced prolonged depolarization afterpotential (PDA) after blue light stimulation. (**B**) Immunocytochemical cross sections through ommatidia of 1 day old wild-type, Rh1::TbID^wt^, and Rh1::TbID^ninaE^ flies. The Rh1 null mutant *ninaE*^*17*^ was used as a negative control. Subcellular localization of Rh1::TbID and native Rh1 in the dark-adapted flies (left side) and after 4 h exposure to blue light (right side) is demonstrated. Rhabdomeric actin was stained with Alexa Fluor 546 conjugated phalloidin (green). Rh1::TbID and native Rh1 were labeled with 4C5 antibody, which was detected by a secondary ALF 680-coupled antibody (red). The overlay of both channels appears yellow. In dark-adapted flies both Rh1::TbID and native Rh1 were localized within the rhabdomere. After 4 h of blue light exposure, vesicles containing Rh1 or Rh1::TbID were detected (arrowheads). Scale bar: 5 µm.

The in vivo application of the TurboID proximity labeling system requires the fusion of a bait protein with the proximity labeling enzyme TurboID. Potentially, this fusion could affect the stability, localization, function as well as protein interactions of the bait protein^18^. Therefore, we performed functional assays to assess whether the TurboID fusion construct is physiologically functional and able to substitute native Rh1. The functionality of Rh1::TbID as a photoreceptor was investigated using electroretinography. Figure 2A shows electroretinograms of 3 days old wild-type and transgenic flies containing or missing native Rh1 (Rh1::TbID^wt^ and Rh1::TbID^ninaE^, respectively). Dark adapted flies were subjected to a single orange light stimulus or to an orange–blue–blue–orange–orange protocol (OBBOO, see “Materials and methods” section). In response to orange light, the photoreceptor cells depolarize and remain depolarized until the end of the stimulus in all fly strains tested (Fig. 2A, left traces). Both transgenic lines as well as the wild type show a prolonged depolarization afterpotential (PDA) after blue light illumination, which arises from the fact that blue light converts a large part of Rh1 rhodopsin to metarhodopsin, which cannot be inactivated fully by arrestin 2^9^ (Fig. 2A, right traces). In Rh1::TbID^ninaE^ flies the PDA is less pronounced compared to Rh1::TbID^wt^ and wild-type flies. This finding is readily explained by the reduced content of rhodopsin observed in these flies. Reduction of the PDA indicates binding of arrestin 2 to activated Rh1::TbID leading to inactivation of most light-activated Rh1::TbID molecules by this interaction. As a result, Rh1::TbID functionally activates the phototransduction cascade and generates a physiological response very similar to native Rh1.

Our electrophysiological results already suggest that Rh1::TbID functions in the rhabdomere, the subcellular compartment where phototransduction takes place. To substantiate this finding we determined the subcellular localization of Rh1::TbID using immunohistochemistry (Fig. 2B). We determined the localization of native Rh1 and Rh1::TbID in flies kept in the dark or in blue light for 4 h, which results in partial rhodopsin internalization^11,12^. A monoclonal anti-rhodopsin antibody (4C5) detected native Rh1 as well as Rh1::TbID. Accordingly, the Rh1-staining in the upper panels of Fig. 2B reveals Rh1, the second row Rh1 and Rh1::TbID and the third row Rh1::TbID localization only. Cross sections of compound eyes shown in Fig. 2B indicated that native Rh1 and Rh1::TbID are exclusively located in phalloidin-stained rhabdomeres of R1-6 photoreceptor cells, when flies were dark-adapted. In flies kept in blue light for 4 h, vesicles containing internalized rhodopsin were observed (arrow heads in Fig. 2B), in addition to rhabdomeral rhodopsin. Rhodopsin internalization was detected for both native Rh1 and Rh1::TbID. The results indicate a physiological anterograde transport of Rh1::TbID to the rhabdomere and also a light-induced internalization of this fusion protein, as previously described for native Rh1^11,12^.

In summary, our results indicate that fusion of Rh1 with TurboID does not affect the localization and function of rhodopsin.

### Turbo-ID proximity labeling identifies arrestin 2 as a rhodopsin interaction partner

**Figure 3:**
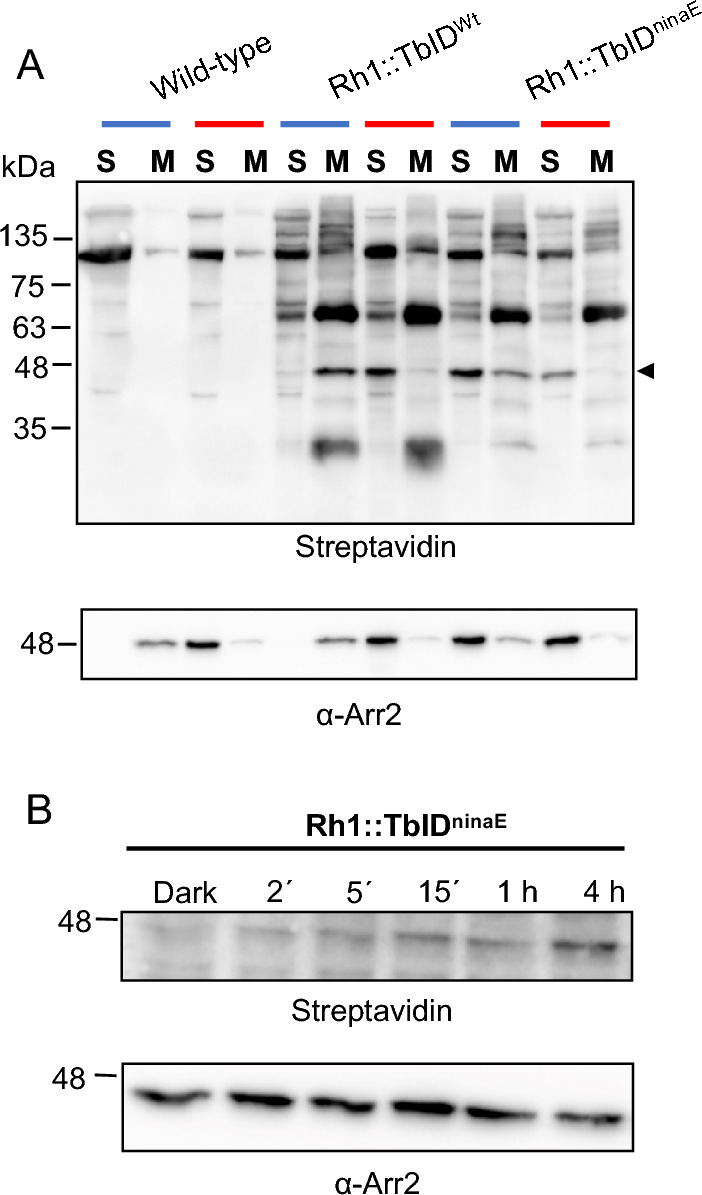
Identification of arrestin 2 as a Rh1 interaction partner by biotin proximity labeling. (**A**) Reversible, light-induced binding of arrestin 2 to photoreceptor membranes of *Drosophila*. 1 day old wild-type, Rh1::TbID^wt^ and Rh1::TbID^ninaE^ flies were exposed to blue light for 4 h (blue bars) to induce arrestin 2 binding to membranes. Part of these flies were then illuminated with red light for 5 min (red bars) to release arrestin 2 from membranes. Protein extracts of soluble proteins (S) and of membrane proteins (M) were loaded on 12% SDS gels (equivalent of 2.4 heads per lane) and blotted after electrophoretic separation. Biotinylated proteins were detected with Streptavidin-HRP (top panel). The arrestin 2 bands are indicated by an arrowhead. (**B**) Time course for biotinylation of arrestin 2 in Rh1::TbID^ninaE^ flies. Flies were kept in the dark for 24 h and then exposed to blue light for 2ʹ, 5ʹ, 15ʹ, 1 h, and 4 h. Protein extracts from heads (equivalent of 2.4 heads per lane) were blotted after electrophoretic separation in 12% SDS gel. Biotinylated proteins were detected with Streptavidin-HRP. Anti-arrestin 2 antibody was used to detect arrestin 2 (bottom panels in **A,B**). The size of molecular weight markers in kDa is indicated on the left. Original full size blots are presented in Supplementary Fig. 4S.

Arrestin 2 binds to activated rhodopsin, metarhodopsin, and shuts off the phototransduction cascade by sterically inhibiting the interaction of rhodopsin with the visual G protein^6,9^. *Drosophila* arrestin 2 has a molecular weight of 48 kDa and is a soluble protein, but it becomes associated with the photoreceptor membrane upon binding to metarhodopsin^19,20^. We utilized this switch of arrestin 2 between the soluble and membrane fraction to evaluate whether the observed biotinylated 48 kDa protein corresponds to arrestin 2. We prepared extracts from fly heads containing either soluble or membrane associated proteins of flies kept in blue light for four hours to generate metarhodopsin, and of flies kept in blue light and then in red light for 5 min, to reconvert metarhodopsin into rhodopsin and to release arrestin 2 from membranes. Extracts were subjected to SDS-PAGE and probed with streptavidin for biotinylated proteins (Fig. 3A). The biotinylated 48 kDa protein was detected in extracts from transgenic flies, Rh1::TbID^wt^ and Rh1::TbID^ninaE^, but not in wild-type controls. In blue light-illuminated flies the biotinylated 48 kDa protein was detected predominantly in the membrane fraction, whereas after red light illumination this protein was released from the membrane and detected mainly in the soluble fraction. As revealed by immunoblot analysis the same distribution was observed for arrestin 2 (Fig. 3A, lower panel). These results clearly indicate that the biotinylated 48 kDa protein is arrestin 2. Since arrestin 2 behaves identical in transgenic Rh1::TbID expressing flies no matter whether native Rh1 is present or not, arrestin 2 binds to the rhodopsin TurboID fusion protein like it does to native Rh1.

The biotinylated 48 kDa protein was detected only when flies were exposed to light at some point. This makes sense, because arrestin 2 interacts with light-activated rhodopsin only. This finding allowed us to determine a time course for arrestin 2 biotinylation and obtain data revealing how fast biotinylation can occur in this system (Fig. 3B). We found that in flies expressing Rh1::TbID, biotinylation of arrestin 2 is already detected after 2 min of blue light illumination. This time scale is in reasonable agreement with data reported for TurboID-mediated biotinylation of proteins in cell culture that was estimated to take 10 min^17^.

In conclusion, the findings reported above provide a proof of principle for our approach, because they show that a well characterized rhodopsin interaction partner becomes biotinylated in the expected light-dependent way.

### Biotinylation assays in *ninaE* and *ninaA* mutants revealed additional biotinylated proteins.

**Figure 4:**
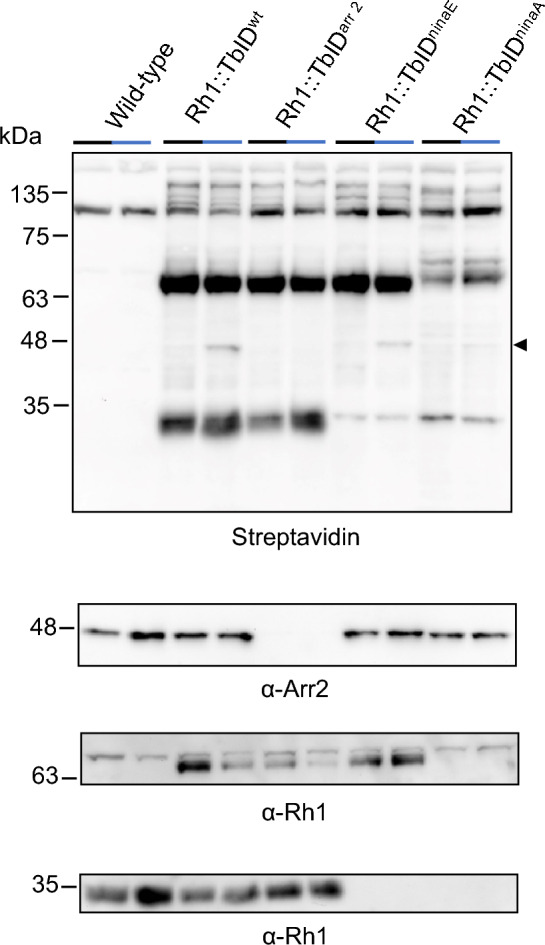
Analysis of biotinylated proteins in *Drosophila* mutants. Western blot analysis of biotinylated proteins with HRP-Streptavidin in heads of wild type, Rh1::TbID^wt^ and *Drosophila* mutants lacking native arrestin 2, Rh1, or NinaA (Rh1::TbID^arr2^, Rh1::TbID^ninaE^, Rh1::TbID^ninaA^, respectively). 1 day old flies raised on medium supplemented with 100 μM biotin were kept in the dark (black bars) or illuminated with blue light for 4 h (blue bars). The lower panels show immunoblots of the same gel probed with α-Arr2 or α-Rh1 antibody. Protein extracts from heads (equivalent of 2.4 heads per lane) were blotted after electrophoretic separation in 12% SDS gel. The size of molecular weight markers in kDa is indicated on the left. Biotinylated arrestin 2 band is reduced in Rh1::TbID^ninaA^ and absent in Rh1::TbID^arr2^ (arrowhead). Original full size blots are presented in Supplementary Fig. 4S.

We crossed the Rh1::TbID transgene into the phototransduction mutants *ninaE*^*17*^, *arr2*^*3*^, and *ninaA*^*2*^. *ninaE*^*17*^ lacks Rh1 rhodopsin^1,2^, *arr2*^*3*^ lacks arrestin 2^7^, and *ninaA*^*2*^ is a null mutation in an endoplasmic reticulum-associated chaperone requited for Rh1 maturation^21^. The *ninaA* mutation results in a vastly diminished amount of mature Rh1 and an elevated amount of immature, glycosylated Rh1^22–24^. Wild-type control and mutant flies were kept in the dark or in blue light and head extracts were probed for biotinylated proteins by streptavidin blots (Fig. 4). In the Rh1 null mutant, *ninaE*^*17*^, most of the heavily biotinylated protein band at 32 kDa was missing, indicating that this protein is Rh1. However, a minor band was still detected at this molecular weight. As immunoblotting revealed no signal corresponding to Rh1 in these samples, we assumed that this minor band is not residual Rh1 but another biotinylated protein that is masked by the Rh1 band in wild-type background. In the *ninaA*^*2*^ mutant a similar band is observed at 32 kDa, but since this mutant probably contains residual amounts of Rh1, we cannot exclude that this band (partially) corresponds to Rh1. In *ninaA*^*2*^ an additional band is detected above the major Rh1::TbID band at ca. 70 kDa. Like native Rh1, Rh1::TbID is also reduced in this mutant as revealed by reduced labeling with streptavidin and a reduced signal on immunoblots probed with anti-Rh1 antibody. As expected, in the *arr2*^*3*^ mutant the biotinylated 48 kDa protein was missing, providing additional proof that this rhodopsin interaction partner is arrestin 2. Biotinylated arrestin 2 is also reduced in the *ninaA*^*2*^ mutant most likely due the largely reduced amount of Rh1::TbID in this mutant.

### Biotinylated proteins in Rh1::TbID flies encompass rhabdomeral and transport proteins

**Figure 5:**
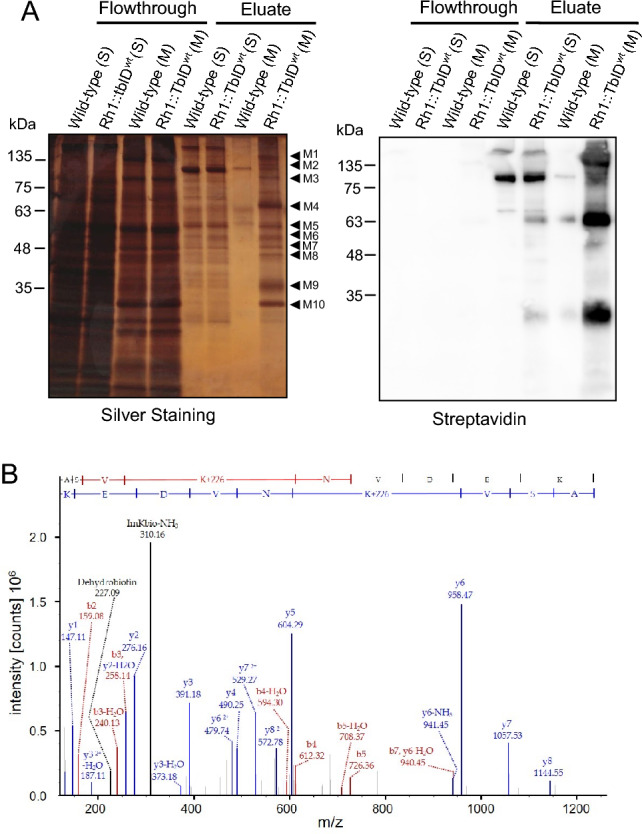
Streptavidin pull down of biotinylated proteins for mass spectrometry analysis. (**A**) Wild-type and Rh1::TbID^wt^ flies were illuminated with white light for 12 h. Protein extracts of soluble proteins (S) and of membrane proteins (M) were incubated with streptavidin beads to pull down biotinylated proteins. Flowthrough and proteins eluted from the beads after pull down were loaded on 12% SDS gels (equivalent of 4.5 and 25 heads per lane for flowthrough and eluate, respectively). One Gel was stained with silver nitrate (left panel) and an equivalent gel was blotted and probed with streptavidin (right panel). Arrowheads indicate protein bands that were excised from lanes Wild-type (M) and Rh1::TbID^wt^ (M) for LC–MS/MS analysis. The size of molecular weight markers in kDa is indicated on the left. (**B**) MS/MS spectrum of the biotinylated peptide ASVKNVDEK from TRP. Biotin is attached to the first lysine. Detected biotin signature fragment ions: dehydrobiotin, 227.09 Da; immonium ion of biotinylated lysine minus NH_3_ (ImKbio-NH_3_), 310.16 Da. Original full size blots and gels are presented in Supplementary Fig. 4S.

Next we asked which proteins besides Rh1::TbID, Rh1 and arrestin 2 become biotinylated in Rh1::TbID flies and thus represent possible Rh1 interaction partners. In order to identify these proteins we purified biotinylated proteins by pull-down with streptavidin-conjugated beads, separated the enriched proteins by SDS-PAGE and performed, in parallel, a silver staining and a streptavidin blot of the gels in order to visualize biotinylated protein bands (Fig. 5, Suppl. Fig. 1S). To further reduce complexity of probes, we initially separated soluble and membrane proteins by differential extraction of proteins from *Drosophila* heads. Most biotinylated protein bands, that were specifically present in the purified sample of Rh1::TbID flies but not in the wild-type control, were observed in the membrane fraction. Corresponding protein bands from the silver-stained gel were excised and subjected to in-gel trypsin digestion. Obtained peptides were analyzed by LC–MS/MS and identified by data base searches of the *Drosophila melanogaster* Uniprot database, allowing protein modifications, such as oxidation of methionine and specifically biotinylation (samples are referred to as TbID^wt^_M1–M10 in Suppl. Table 1S). As control, the corresponding gel slices were cut out from the lane containing enriched proteins of the wild-type flies (samples are referred to as wt_M1–M10 in Suppl. Table 1S). Using highly stringent criteria (i.e. identification of proteins by at least five peptides and a ≥ threefold higher number of identified peptides in the Rh1::TbID flies compared to controls) we identified about 150 potential rhodopsin interaction partners. A selection of the results is depicted in Table 1, all identified proteins are shown in the Supplementary Material (Suppl. Table 1S). As an estimate for the abundance of each identified protein, Table 1 lists the number of identified peptides for each of these proteins obtained under the experimental conditions described above. This analysis was performed twice and only those proteins consistently identified were considered. In addition, we performed an LC–MS/MS analysis with purified biotinylated proteins that were not separated by SDS-PAGE but subjected to trypsin digestion while still bound to streptavidin beads. This analysis was carried out in triplicate for soluble and membrane bound proteins. Results, including a statistical evaluation (volcano plots), are provided in the Supplementary Material (Suppl. Table 2S, Suppl. Fig. 2S). Notably, 26 of the 38 proteins listed in Table 1 are statistically significant up-regulated in this experiment as well (indicated by “+” in Table 1). When comparing the two approaches, that is digestion of excised protein bands and on-bead digestion, we find the first approach more reliable. As also observed in other publications, on-bead digestion resulted in many unspecific hits including proteins that are up-regulated in the control (e.g.^25^). Such unspecific hits detected more frequently in the control were hardly observed when specific protein bands, shown to actually contain biotinylated proteins via streptavidin blots, were excised and analyzed, except for a few proteins that are endogenously biotinylated like pyruvate carboxylase. Accordingly, we focused the evaluation of our results on the gel-based approach. While many proteins are highly enriched in samples obtained from Rh1::TbID expressing flies, the endogenously biotinylated enzyme pyruvate carboxylase is detected with a higher number of identified peptides in wild-type than in Rh1::TbID samples. This serves as an internal control indicating that biotinylated proteins were purified also from wild-type flies when present.

**Table 1:** Streptavidin binding proteins in Rh1::TbID flies.

Protein	Peptides in control	Peptides in probe	Peptide ratio probe/control	On-bead digestion	Accession number
Phototransduction					
TRP	0.5	135.5	271	+	P19334
Arr2	20.5	122.5	6.0	+	A8E775
Rh1	5	72	14.4	+	P06002
PLCβ (NorpA)	4.5	51.5	11.4	+	E1JJD5
CalX	0	47.5	∞	+	Q9VDG5
Arr1	4.5	33	7.3	+	M9NES0
RdgB	4	25.5	6.4	+	M9PJM8
TRPL	0	25	∞	+	P48994
CdsA	0	20.5	∞	+	H9ZJM9
InaD	0	13.5	∞	+	Q24008
RdgA	0	9	∞	+	M9MSN3
ePKC (InaC)	0	7.5	∞	+	P13677
Gαq	4	17	4.3		P23625
Gβ76C	1.5	7.5	5.0		P29829
PIP4K	0	7	∞		Q8SXX1
Rhabdomere structure					
Prominin	1	95.5	95.5	+	B7YZQ3
Chaoptin	9	40.5	4.5		F0JAL7
NinaC	10	39	3.9		P10676
Protein maturation, transport, degradation					
Flotillin 1	4.5	33.5	7.4	+	O61491
Flotillin 2	8	24.5	3.1	+	M9PJN2
PIP82	0.5	31	62	+	Q9W3E2
Vap33	0.5	28	56	+	Q9W4N8
Rab32	4.5	26	5.8	+	A1Z7S3
Rbcn-3B	0	10.5	∞		O46092
Calnexin 99A	0.5	9.5	19	+	Q0KHZ9
EMC1	0	6	∞		Q9VHY6
Stardust	0	7	∞	+	X2JCZ1
Clathrin	5.5	17.5	3.2		P29742
AP2 complex subunit alpha	3	13.5	4.5		P91926
AP complex subunit beta	3	9.5	3.2		Q24253
Dynamin (Shi)	7	17	2.4		E1JJA4
Sec63	0	6.5	∞	+	Q9VS57
Others					
Calcium-transporting ATPase	3	25	8.3	+	Q9V4C7
Oxysterol Binding Protein Orp8	0	18	∞	+	B7Z0M9
Gαo	4	19	4.8	+	P16378
Gαs	1.5	10	6.7	+	A0A0B4LGI1
Kazachoc (kcc)	1	14.5	14.5	+	A0A0B4LGD3
Endogenously biotinylated proteins					
Pyruvate carboxylase	126	83.5	0.7		Q0E9E2

Selected proteins that were identified by LC–MS/MS in two experiments from excised silver-stained protein bands using stringent criteria (Protein Threshold: 99.9%, Minimal Number of Peptides: 5, Peptide Threshold: 95%) are listed. The indicated number of peptides is the average from two independent experiments. “+” in the column “on-bead digestion” indicates that this protein was found to be up-regulated significantly in the on-bead digestion experiment also.

To enrich experimental evidence, we purified and analyzed biotinylated proteins from fly heads expressing the Rh1::TbID transgene in *ninaE*^*17*^ and *ninaA*^*2*^ background (Suppl. Fig. 1S). Additional protein bands, masked by the Rh1 band or not present in Rh1::TbID^wt^, were detected in these samples (TbID^ninaE^_M9 and TbID^NinaA^_M11), excised and subjected to LC–MS/MS. Proteins detected exclusively in these samples are depicted in Table 2. Of note, biotinylated peptides were identified in eight proteins, most prominently in TRP which contained seven different biotinylated peptides. The identification of biotinylated peptides by LC–MS/MS is in agreement with specific purification of biotinylated proteins in samples obtained from TurboID-expressing flies (Table 3). A MS/MS spectrum obtained from a biotinylated TRP peptide revealing biotin signature fragment ions^26^ is shown as an example in Fig. 5B. Other spectra of biotinylated peptides are shown in Suppl. Fig. 3S.

**Table 2:** Proteins found in the *ninaA* mutant only.

Protein	Number of peptides	Accession number
DnaJ homolog subfamily C	9	Q7PLG1
E3 UFM1-protein ligase 1 homolog	7	A0A0B4K6K7
Pkc53E	6	A0A0B4KFT3
Inner nuclear membrane protein Man1	5	Q7JRE4

Proteins that were detected with ≥ 5 peptides only in the excised band from Rh1::TbID^ninaA^.

**Table 3:** Proteins containing identified biotinylated peptides.

Protein	Number of bio-tinylated peptides	Observed in samples	Accession number
TRP	7	1	P19334
Rh1	2	1–10	P06002
Prominin	1	1, 2	B7YZQ3
PLCβ (NorpA)	1	2, 4	E1JJD5
CdsA	1	8	H9ZJM9
Oxysterol-binding protein Orp8	1	2	B7Z0M9
DnaJ homolog subfamily C	1	11	Q7PLG1
Vesicle transport protein USE1	1	10	Q9VSU7

Biotinylated peptides (Scaffold peptide threshold: 90%) were identified in the following samples: 1 = TbIDwt_M1, 2 = TbIDwt_M2, 3 = TbIDwt_M3, 4 = TbIDwt_M4, 5 = TbIDwt_M6, 6 = TbIDwt_M6, 7 = TbIDwt_M7, 8 = TbIDwt_M1, 9 = TbIDwt_M9, 10 = TbIDwt_M10, 11 = TbIDninaA_M11.

As compared to the wild-type control, proteins highly enriched in the Rh1::TbID^wt^ sample include phototransduction proteins such as the ion channels TRP and TRPL, *norpA*-encoded phospholipase Cβ, both *Drosophila* arrestins (Arr1 and Arr2), and the alpha and beta subunit of the visual G protein (Gαq, Gβ76C). Most abundantly observed among these phototransduction proteins and directly shown to be biotinylated by identification of biotinylated peptides was the TRP ion channel. Rh1 has been proposed to be part of a signal transduction complex (signalplex) assembled by the scaffolding protein INAD, that also comprised TRP, PLCβ and a protein kinase C (ePKC)^27^. TRPL was also suggested to be part of the signalplex via heteromultimerization with TRP^28^. However, while the assembly into a signaling complex of TRP, PLCβ and ePKC by INAD is generally accepted, the binding of significant amounts of Rh1 to the INAD signaling complex as well as formation of TRP/TRPL heteromultimers is not supported by other studies^29–31^. The scaffolding protein INAD and the protein kinase C (ePKC), were also detected in the Rh1::TbID^wt^ sample, albeit at a relatively low abundance. The identification of TRP, TRPL, NorpA, ePKC, and INAD as possible Rh1 interaction partners could be explained with the existence of a proposed signalplex containing all of these components. However, given that the radius of TurboID-mediated biotinylation is estimated to be ca. 10 nm^17^, it cannot be excluded that presence of Rh1::TbID in the narrow space of rhabdomeral microvilli, that have a length of about 2 µm and a diameter of 50 nm, results in biotinylation of abundant rhabdomeral membrane proteins without the need of permanent protein–protein-interaction. This may include proteins involved in phosphoinositide metabolism, that is RdgA, RdgB, CdsA, and PIP4K, the Na/Ca exchange protein CalX, and structural proteins of the rhabdomere, namely Prominin, Chaoptin, and NinaC, which were detected as possible Rh1 interaction partners. Prominin and Chaoptin are part of a complex required for formation of an open rhabdom^32,33^, while NinaC attaches the INAD signaling complex to the rhabdomeral actin cytoskeleton^34^. The biotinylation of these rhabdomeral proteins may result from diffusion of Rh1::TbID in the rhabdomeral membrane leading to transient interactions. Alternatively, activated biotin (Biotin-5ʹ-AMP) may reach high concentrations within rhabdomeral microvilli and attach biotin to rhabdomeral proteins without the need of a direct protein–protein interaction. The latter possibility would constitute a severe limitation for detecting protein–protein interactions in the rhabdomeral microvilli (or any other tiny spaced subcellular compartment) by this method. Our finding that arrestin 2 becomes biotinylated only after rhodopsin is activated by light argues against unspecific biotinylation of rhabdomeral proteins, because the generation of Biotin-5ʹ-AMP by TurboID should be light-independent. It should be noted, however, that a significant fraction of arrestin 2 is translocated from the cell body to the rhabdomere upon illumination, thus enhancing the amount of arrestin 2 in the rhabdomere^35,36^. Attaching TurboID to an immobilized rhabdomeral protein such as NinaC, which is bound to the microvillar actin cytoskeleton, may help to distinguish between these alternatives.

The very high biotinylation of TRP could alternatively result from common vesicular transport of Rh1 and TRP from the ER to the rhabdomere^37^. Likewise, Rh1 and TRPL were shown to be present in the same endocytic vesicles following light-triggered internalization of both proteins^38^. In addition to rhabdomeral proteins we observed ribosomal proteins and elongation factors presumably interacting with Rh1 during protein translation. We also detected numerous mitochondrial proteins, but the significance of this remains to be uncovered.

Importantly, several proteins linked to protein maturation and transport were identified as potential interaction partners of Rh1. These include Calnexin 99A, EMC1, Rab32, Vap33 and Rbcn. The ER chaperon Calnexin 99A is a transmembrane protein that was reported to assist Rh1 maturation^39^. The ER Membrane Protein Complex 1 (EMC1) is required for biosynthesis of multi-pass membrane proteins, including Rh1^40^. This requirement may be indirect as EMC1 was reported to be needed for import of the rhodopsin chaperon Xport-A into the ER membrane rather than for Rh1 biosynthesis directly^41^. However, our results support a direct interaction of Rh1 with EMC1. Another important rhodopsin chaperone, NinaA, was identified with two peptides in the in-gel digests and with three peptides in the on-bead digests of samples obtained from Rh1::TbID flies but not in the control (see Excel files provided in the Supplement). It is not included in Table 1, because it failed our stringent criteria (identification with at least five peptides), which in this case may be due to the small size of NinaA (26 kDa) or less efficient biotinylation of this protein.

The Rab proteins, Rab 1, Rab 5 and Rab 11, have previously been shown to be involved in Rh1 transport or endocytosis^38,42–44^. Rab 32, which has been identified in our study, may represent a novel Rh1-interacting Rab protein required for either anterograde or retrograde rhodopsin transport. VAMP-associated protein 33 kDa (Vap33) has been described to control synaptic growth and axonal transport^45^, but may be involved in more general vesicular transport processes including rhodopsin transport. Another protein involved in Golgi organization and protein secretion is the vesicle transport protein USE1 that we found to be biotinylated (Table 3)^46^. Rh1 is assumed to be incorporated into the rhabdomere via the apical stalk membrane, a part of the plasma membrane located between the rhabdomere and membrane adherence junctions, by a Crumbs and Myosin V-dependent transport^47^. Accordingly, identified proteins PIP82 and Stardust, which are located in this region, may interact with Rh1 during its incorporation into the apical membrane^48,49^.

Concerning rhodopsin internalization, it has been reported that activated Rh1 is removed from the rhabdomere by Clathrin-mediated endocytosis^12,50,51^. The identification of Clathrin, Dynein and subunits of the AP-adapter complex are in agreement with Clathrin-mediated endocytosis. Interestingly, Flotillin 1 and 2 were also identified as possible Rh1 interaction partners. Flotillins are associated with caveolae^52^, raising the possibility of a second Rh1 internalization route via caveolae. Rabconnectin-3B (Rbcn) encodes a protein that is part of a regulatory subunit of the vacuolar H^+^ ATPase required for acidification of intracellular vesicles and the lysosome. It was shown to function in endocytosis of Notch and Notch signaling^53^. Its proposed interaction with Rh1 may suggest a requirement for lysosomal degradation of endocytosed Rh1. Finally, among other potentially interesting Rh1 interaction partners detected in Rh1::TbID^wt^ flies are a plasma membrane calcium transporting ATPase (PMCA), other G protein alpha subunit (Gαo, Gαs), and a potential phospholipid transport protein, Orp8, which so far have not been discussed in the context of photoreception. For Orp8 a biotinylated peptide was detected in the LC–MS/MS analysis providing additional support for direct interaction with Rh1::TbID (Table 3).

In the *ninaA*^*2*^ mutant, Rh1 fails to fold correctly, is not transported to the rhabdomere and becomes degraded^22–24^. Proteins detected exclusively in the Rh1::TbID^ninaA^ sample include a DnaJ homolog subfamily C, a UFM1-protein ligase and the protein kinase C Pkc53E (Table 2). DnaJ proteins are co-chaperons of HSP70 proteins that may assist folding of misfolded Rh1 or mediate Rh1 degradation. A human DnaJB12 mediates selective ER-associated autophagy of P23H-rhodopsin, a mutated rhodopsin causing Retinitis Pigmentosa^54^. E3 UFM1-protein ligase mediates ufmylation of target proteins. Ufmylation is a recently identified small ubiquitin-like modification, whose biological function and relevant cellular targets are poorly understood^55^.

### Mutations in putative Rh1 interaction partners can result in a reduced Rh1 content

**Figure 6:**
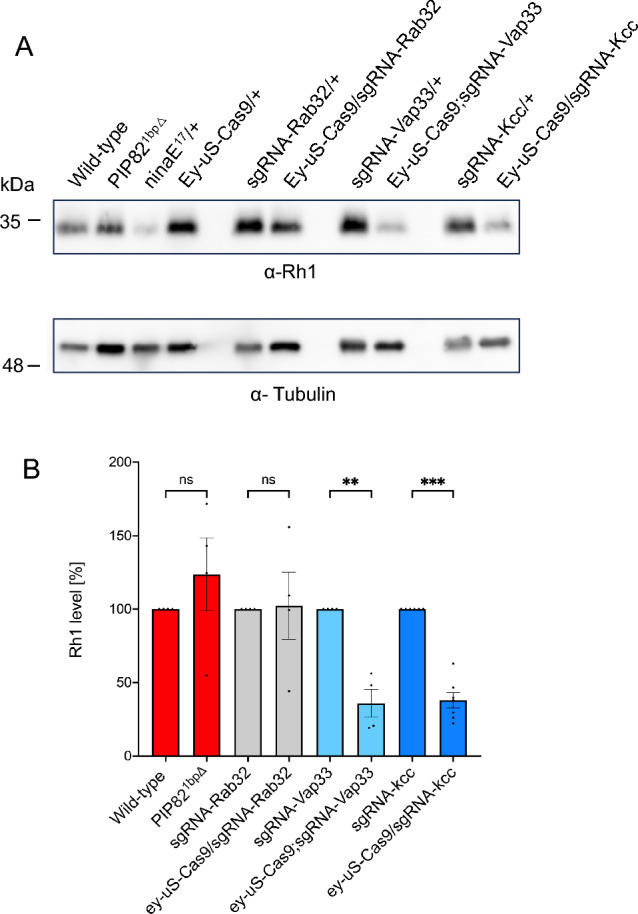
Quantitative immunoblot analysis of the Rh1 content in selected mutants. Head extracts of the PIP82^1bp∆^ and tsCRISPR-induced mutants for Rab32, Vap33 and Kazachoc (kcc) were probed with the monoclonal Rh1 antibody and an anti-Tubulin antibody to assess Rh1 amounts. Wild-type flies were used as a control for PIP82^1bp∆^, as controls for tsCRISPR mutants the respective sgRNA lines without induction of sgRNA expression were used. (**A**) Shows a representative immunoblot. Upper panel was probed anti-Rh1 antibody, lower panel was probed with anti-Tubulin antibody, which was used as a loading control. (**B**) Quantification of Rh1 in indicated mutants vs. control flies. Rh1 signals were normalized with Tubulin signals and compared to the respective controls on the same blot that were set to 100% (n = 4 for PIP82^1bp∆^, Rab32, Vap33; n = 7 for Kazachoc; error bar: SEM; **p < 0.002; ***p < 0.0002; *ns* not significant). Original full size blots are presented in Supplementary Fig. 4S.

In order to asses a possible functional role of selected putative Rh1 interaction partners for rhodopsin stability, we tested available mutants for the proteins PIP82, Rab32, Vap33 and Kazachoc for changes in the amount of Rh1 in *Drosophila* heads. The mutant PIP82^1bp∆^ is a previously described null mutant^48^, Rab32, Vap33 and Kazachoc mutants were generated using tsCRISPR, by crossing an eyless-Gal4 driver line with the respective sgRNA lines. Using quantitative immunoblot analysis, we observed wild-type amounts of Rh1 in PIP82 and Rab32 mutants, but a reduced rhodopsin content in Vap33 (36% of control) and Kazachoc (38% of control) (Fig. 6). For PIP82^1bp∆^ the same result, that is no change in the rhodopsin content, has previously been described by Zelhof et al.^48^. However, this mutant revealed partial misdirection of Rh1 and other rhabdomeral proteins to the basolateral membrane rather than to the base of the rhabdomere. The reduced Rh1 contents in Vap33, a possible transport protein, and Kazachoc, a potassium-chloride cotransporter^56^, may indicate defects in rhodopsin stability in mutants devoid of these potential interaction partners.

## Conclusion

**Figure 7:**
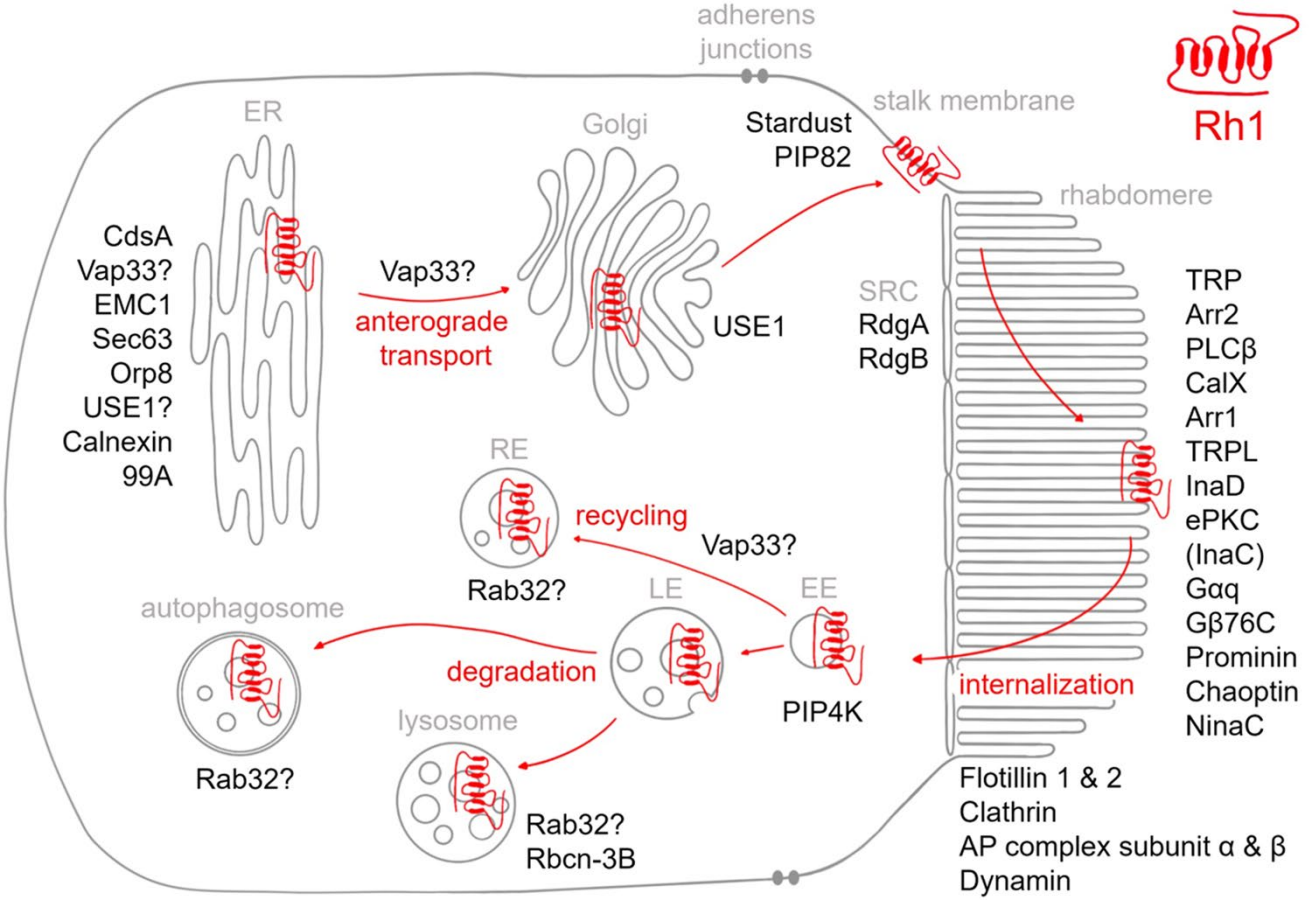
Scheme of a *Drosophila* photoreceptor cell showing localization of putative Rh1 interaction partners. Arrows indicate anterograde and retrograde transport routes of Rh1. *ER* endoplasmic reticulum, *Golgi* Golgi apparatus, *SRC* subrhabdomeric cisternae, *EE* early endosome, *RE* recycling endosome, *LE* late endosome.

In this study, we employed biotin proximity labeling to identify possible interaction candidates of the major *Drosophila* rhodopsin Rh1. Our investigation revealed almost all recognized proteins essential for phototransduction or for the structural integrity of the rhabdomere that are located in or associated with the rhabdomeral membrane. In addition, we established that arrestin 2, an abundant soluble protein in the eye, only becomes biotinylated in light-conditions, in which it relocates from the cell body to the rhabdomere and interacts with Rh1^35,36^. Assuming an approximate radius of 10 nm for Turbo-ID-mediated biotinylation, we conclude that rhabdomeral Rh1 comes into direct contact with most other proteins of the rhabdomeral membrane. Our study also uncovered proteins that function in protein folding in the ER, vesicular protein transport, endocytosis, and protein degradation. These identified proteins include both previously described Rh1 interaction partners as well as hitherto unrecognized potential interaction candidates. The assumed subcellular localization in a *Drosophila* photoreceptor cell of all putative Rh1 interaction partners discussed here is indicated in Fig. 7. The newly discovered candidate proteins open promising perspectives for a better understanding of rhodopsin maturation and transport in future investigations. Finally, putative Rh1 interaction partners detected only in the *ninaA*^*2*^ mutant suggests a potential degradation pathway via ER-associated autophagy for misfolded Rh1 in the absence of NinaA. A similar degradation pathway has been described for misfolded human rhodopsin that causes Retinitis Pigmentosa^54^. Accordingly, studies of this degradation pathway in *Drosophila* may further our knowledge and offer novel intervention points to treat degenerative eye diseases.

## Materials and methods

### Fly husbandry and illumination conditions

Flies were raised and kept on yeast/cornmeal food (84 g/l fresh baker’s yeast, 50 g/l cornmeal, 32 g/l sucrose, 12 g/l agar, supplemented with methylparaben, vitamin C and propionic acid) at 25 °C. For biotin supplementation, adult flies were kept on yeast/cornmeal food supplemented with 100 μM Biotin.

The following fly mutants were used: Rh1 null mutant: y w;; *ninaE*^*17*^; Arr2 null mutant: w; *arr2*^*3*^; NinaA null mutant: w; *ninaA*^*2*^; PIP82 null mutant: PIP82^1bp∆^^48^; sgRNA Vap33: Bloomington *Drosophila* Stock Center (BDSC) #81751; sgRNA Rab32: Vienna *Drosophila* Resource Center #342622; sgRNA Kcc: BDSC # 91934; ey driver line: P[GMR-w.IR]13D/FM7; ey > Gal4 UAS > uS-Cas9/CyO.

For dark-adaptation, flies were kept in the dark for the indicated period at 25 °C. For illumination, flies were kept under a white, fluorescent tube (4000 K, 1750 Lux, E_e_^470 nm^ = 298 μW/cm^2^, E_e_^590 nm^ = 215 μW/cm^2^, Osram, Munich, Germany) at room temperature. For studying arrestin 2 binding to membranes, flies were illuminated with blue light for 4 h in a blue transparent plastic box (310–490 nm, E_e_^470 nm^ = 28 μW/cm^2^) and dissected immediately or were illuminated first with blue light for 4 h and then with red light for 5 min using a red LED (660 nm, Roithner Lasertechnik, Vienna). For immunocytochemistry flies were either kept in the dark for 24 h or illuminated with blue light for 4 h in a coloured transparent plastic box under a fluorescent light tube (310–490 nm, E_e_^470 nm^ = 28 μW/cm^2^).

Dark-adapted flies or red light illuminated flies were prepared for subsequent experiments using a weak cold light source (KL 1500 LCD, Schott, Mainz, Germany) with a deep red long pass filter (RG630, Schott). Light-adapted flies were prepared under blue light or in white room light, respectively.

### Cloning and expression of Rh1::TbID

For generating a Rh1::TbID construct, we used a cDNA clone as a template for PCR amplification of *rh1.* The 3ʹ-primer was modified to remove the *rh1* stop codon and to introduce a EcoRI restriction site. The modified *rh1* cDNA was digested with BamHI and EcoRI and was cloned into pENTR^TM^1A (a vector containing attL sites for site-specific recombination; Invitrogen, Germany). Next, turboID-V5 cDNA was PCR-amplified from a commercially available plasmid vector (pCDNA3-TurboID; Addgene #107173) using primers to introduce an EcoRI site at 5ʹ and an XhoI site at 3ʹ of the cDNA. After digestion with EcoRI and XhoI, the turboID cDNA was cloned in frame into the pENTR TM 1A plasmid containing the *rh1* cDNA. A nine amino acid linker was added between Rh1 and TbID.

The construct in the pENTR^TM^1A vector, was transferred into a modified YC4 vector by site specific recombination of att sites using the Gateway® technology from Invitrogen (Germany). The modified YC4 vector contained the *Drosophila Rh1*-promoter (base pairs − 833 to + 67), attR-sites and the last 0.6 kb of the 3ʹ-untranslated region of *Rh1*^57^. For cloning, PCR fragments were amplified using Q5 polymerase (New England BioLabs (NEB)). The vectors were double digested using standard enzymatic restriction digest and ligated to gel-purified PCR products by T4-DNA ligation. Ligated plasmid products were introduced by heat shock transformation into competent XL1-Blue bacteria. All clones were verified by DNA sequencing.

P-element-mediated transformation of the host strain *Drosophila yellow white* (*yw)* was carried out by *Drosophila* injection service at FlyORF (Zurich).

A transgenic line with the transgene on the third chromosome (yw; P[y^+^;; rh1 > Rh1::TbID]) was used in experiments. Flies expressing Rh1::TbID in *ninaA*^*2*^, *ninaE*^*17*^ and *arr2*^*3*^ mutant background were generated by using standard *Drosophila* crossing schemes to obtain: yw; ninaA^2^; P[y^+^, rh1 > Rh1::TbID], yw; P[y^+^, rh1 > Rh1::TbID]; ninaE^17^, and yw; P[y^+^, rh1 > Rh1::TbID]; arr2^3^. The strain *Drosophila yellow white (yw*) was used as a control and is referred to as “wild-type” in this study.

### Extraction of proteins from fly heads

For whole-head protein extractions, 10–20 fly heads were homogenized in 4 μl SDS extraction buffer per head (4% (w/v) SDS, 1 mM EDTA, 75 mM Tris/HCl, pH 6.8) and incubated for 10 min at room temperature. Extracts were centrifuged at 14,000×*g* for 10 min at room temperature, and the supernatant was mixed with 0.2 volumes of SDS sample buffer (5% (w/v) SDS, 30% (v/v) glycerol, 5% (v/v) 2-mercaptoethanol, 100 μg/ml bromophenol blue, 500 mM Tris/HCl, pH 6.8).

For assessing arrestin 2 binding to membranes 100 fly heads per genotype and light condition were homogenized in 100 μl lysis buffer (50 mM Tris–HCl, pH 7,5; 150 mM NaCl; 50 µM p-Amidinophenyl-methanesulfonyl fluoride (APMSF)) while kept on ice and then centrifuged at 100,000×*g* for 10 min (4 °C). The supernatant (containing cytosolic proteins) was aspirated, the pellet was washed in lysis buffer and resuspended in 100 μl lysis buffer containing Triton X-100 (50 mM Tris–HCl, pH 7,5; 150 mM NaCl; 50 µM APMSF; 1% Triton X-100). The resuspended pellet was centrifugated at 100,000×*g* for 10 min (4 °C). The supernatant (containing membrane proteins) was transferred to a fresh tube. Before loading the samples on SDS-PAGE they were supplemented with 0.2 volumes sample buffer for soluble proteins (20% (w/v) SDS, 5% (v/v) 2-mercaptoethanol, 100 μg/ml bromophenol blue, 500 mM Tris/HCl, pH 6.8).

### Immunoblot and steptavidin blot analysis

For immunoblotting, 15 μl of the samples were loaded onto 10% or 12% polyacrylamide gels, separated by SDS-PAGE, and transferred to a PVDF-membrane (Bio-Rad, Hercules, CA). Membranes were blocked for 30 min in TBS-T with 5% skim milk, 0.1% Tween 20 (150 mM NaCl, 10 mM Tris/HCl, pH 7.5) for immunodetection with monoclonal Rh1 antibody (DSHB Cat# 4C5, RRID: AB_528451) and anti-Tubulin antibody (DSHB Cat# E7, RRID: AB_528499), or overnight in TBS-T with 5% BSA for streptavidin-HRP staining and immunodetection with rabbit anti-Rh1 antibody.

After blocking, membranes were incubated with primary antibody in TBS-T with 5% skim milk overnight at 4 °C. For incubation with Streptavidin-HRP (Thermo Fischer Scientific, Cat# N100) 1:1000, and rabbit anti-Rh1 membranes were incubated in TBS-T with 5% BSA for 2 h at room temperature. Antibodies were used at the following concentrations: rabbit anti-Rh1^58^, 1:1000, rabbit anti-V5 (Thermo Fischer Scientific, Cat# A190-120A), 1:1000, rabbit anti-arr2^59^, 1:1000, and mouse anti-Tubulin (DSHB Cat# E7, RRID: AB_528499) 1:1000. PVDF-membranes were then subjected to three washings with TBS-T for 5 min each and thereafter incubated with secondary antibody in TBS-T with 5% skim milk for 1 h at room temperature. HRP-conjugated secondary antibodies were used 1:10,000 (Sigma-Aldrich, St. Louis, MO; Cat# A0545, A9044, RRID: AB_257896, AB_258431). After three final washing steps in TBS-T, 5 min each, signals were detected by enhanced chemiluminescence (0.091 M Tris/HCl pH 8.6; 0.0227% (w/v) luminol; 0.01% (w/v) para-hydroxycoumaric acid; 0.01% H_2_O_2_) with the ChemiDoc XRS + Molecular Imager using the software Quantity One 4.6.9 (Bio-Rad).

### Quantification of immunoblot signals and statistics

Quantification of immunoblot signals was performed with Image Lab 4.0 (Bio-Rad) by integration of the pixel intensities of each protein band. Rh1 and Rh1::TbID signals were normalized using Tubulin signals of the same sample. Values obtained for Rh1 in wild-type flies were set to 100%. Six biological replicates were analysed per data point. Statistical analyses were conducted using GraphPad Prism 10.0.2 (GraphPad Software, San Diego, CA; RRID: SCR_002798). Evaluations between two means were performed as a one-way ANOVA with Tukey or Bonferroni post-hoc test. Statistical significances are indicated as part of the resulting graphs in the form of horizontal bars connecting two columns according to their p-values (ns, not significant; **p < 0.002; ***p < 0.0002).

### Immunostaining of fly eyes and fluorescence microscopy

Sample preparation for conventional fluorescence microscopy was conducted as described^60^: *Drosophila* fly heads were separated from the body and fixed in 2% (w/v) paraformaldehyde (PFA) in PBS (137 mM NaCl, 2.7 mM KCl, 10 mM Na_2_HPO_4_, 2 mM KH_2_PO_4_, pH 7.2) for 45–60 min at room temperature. Heads were washed twice for 10 min with phosphate buffer (77 mM Na_2_HPO_4_, 23 mM NaH_2_PO_4_, pH 7.4) and then dehydrated in two steps with sucrose, first with 10% (w/v) sucrose, followed by 25% (w/v) sucrose in phosphate buffer at room temperature for 45 min and 30 min, respectively. Finally, the eyes were dehydrated with 50% (w/v) sucrose in phosphate buffer overnight at 4 °C and embedded in Tissue-Tek O.C.T. (Sakura, Tokyo, Japan). Cryosections of 10 μm thickness of *Drosophila* eyes were obtained at minus 25 °C using a CM3050 S Cryostat (Leica, Wetzlar, Germany). Slices were fixed in 2% (w/v) PFA in PBS for 5–10 min at room temperature and then washed three times in PBS, for 5 min each. For immunostaining, slices were blocked in PBS-T (1% (w/v) BSA, 0.3% (v/v) Triton X-100 in PBS) for 2 h at room temperature. After blocking, sections were incubated with primary antibody (mouse anti-Rh1 (DSHB Cat# 4C5, RRID: AB_528451), 1:50) in PBS-T overnight at 4 °C.

Sections were subsequently washed three times with PBS, for 5 min each, and thereafter incubated with secondary antibody in PBS-T for 2 h at room temperature. An AF 660-conjugated secondary antibody was used 1:100 (Thermo Fisher Scientific, Waltham, MA**,** Cat# A-21054, RRID: AB_2535721) and AF 546-conjugated phalloidin (Thermo Fisher Scientific Cat# A-22283, RRID: AB_2632953) was added 1:100 to the secondary antibody solution for staining of F-actin in rhabdomeres. After three final washing steps in PBS, 5 min each, washing solution was removed and slices were mounted in Mowiol 4–88 (with 2% n-propyl gallate as anti-fading agent).

For analysis of slices by fluorescence microscopy, images were acquired with an AxioImager.Z1m microscope (objective: EC Plan-Neofluar 40×/1.3 Oil) using the ApoTome module (Carl Zeiss, Jena, Germany). Images were captured with the Axiocam 530 mono (Carl Zeiss) camera using the ZEN 2 (blue edition) software (Carl Zeiss).

### Electroretinography

For ERG recordings, flies were briefly immobilized on ice and fixed inside of improvised yokes made from 200 μl pipette tips before they were mounted in the center of a Faraday cage. Chlorinated silver wires were inserted into glass micropipettes filled with Davenport solution (100 mM NaCl_2_, 2 mM KCl, 1 mM CaCl_2_, 1.8 mM NaHCO_3_, pH 7.2) and utilized as electrodes. Glass micropipettes were pulled from capillaries BF150-75-10 (Sutter Instrument, Tuttlingen, Germany) using a PC-10 puller (Narishige Scientific Instrument Lab., Tokyo, Japan). Silver wires with 250 μm diameter (Good Fellow, Huntingdon, UK) were chlorinated with an ACl-01 automatic chlorider (NPI Electronics, Tamm, Germany). The recording electrode was inserted into the eye just below the cornea and the reference electrode into the head capsule. ERG recordings were performed at room temperature after 3 min of dark adaptation. Electrodes had a resistance of ca. 30 mΩ when immersed in a 0.9% NaCl solution.

Light stimuli lasting 5 s were generated by a PLED-02M (NPI Electronics) driven orange light emitting diode (590 nm, Roithner Lasertechnik, Vienna, Austria) and a blue light-emitting diode (470 nm, Roithner Lasertechnik, Vienna, Austria) in a setup of two collimating lenses (Linos, Göttingen, Germany) within the light path. Two different stimulus protocols were used: OBBOO (5 s orange light, 10 s dark, 5 s blue light, 10 s dark, 5 s blue light, 10 s dark, 5 s orange light, 10 s dark, 5 s orange light) and single orange (5 s orange light, 30 s dark).

The light intensity was determined to be at 3.58 mW cm^−2^ at the point where the fly eye would be. An EXT 10-2F amplifier (NPI Electronics) was used with a 700 Hz low pass filter. Data recording was achieved by whole cell analysis software WinWCP V4.7.6 (University of Strathclyde, Glasgow, Scotland; RRID: SCR_014713).

### Enrichment of biotinylated proteins by streptavidin pulldown

Membrane and cytosolic proteins were extracted from approximately 60 heads of 3-day-old adult flies exposed to white light for 12 h. For the separation of membrane and cytosolic proteins heads were homogenized in 60 μl lysis buffer (50 mM Tris–HCl, pH 7.5; 150 mM NaCl; 50 µM APMSF) while kept on ice and then centrifuged at 14,000×*g* for 10 min (4 °C). The supernatant (containing cytosolic proteins) was aspirated, the pellet was washed in lysis buffer and resuspended in 60 μl RIPA buffer (50 mM Tris pH 8, 150 mM NaCl, 0.1% SDS, 0.5% sodium deoxycholate, 1% Triton X-100 and 50 µM APMSF). The resuspended pellet was centrifugated at 14,000×*g* for 10 min (4 °C). The supernatant (containing membrane proteins) was transferred to a fresh tube. For pulldown assays, streptavidin beads (1 μl of magnetic beads (Thermo Fischer Scientific, Pierce™ Streptavidin Magnetic Beads) per 8 μg of protein) were washed two times for 5 min and equilibrated for 1 h in RIPA buffer, before 200 μg of protein, diluted to 100 μl, was added to the beads. Beads and cell lysates were incubated under constant agitation overnight at 4 °C to allow binding. The beads were subsequently washed twice with 300 µl of RIPA buffer, once with 1 ml of 1 M KCl, once with 1 ml of 2 M urea in 10 mM Tris–HCl (pH 8.0), and twice with 300 µl RIPA buffer. Biotinylated proteins were then eluted by boiling the beads for 5 min at 95 °C in 30 μl of elution buffer (4% (w/v) SDS, 1% β-mercaptoethanol, 100 mM Tris/HCl, pH 6.8; 20 mM DTT; 2 mM biotin). 15 µl of the eluted proteins were separated on a 12% SDS-PAGE gel.

Following SDS-PAGE, the proteins in the gel were visualized using a mass-spectrometry compatible silver stain. All incubation steps were done with shaking on a horizontal shaker at room temperature. First, the gel was incubated overnight in 100 ml of fixative solution (30% (v/v) ethanol 10% (v/v) acetic acid). Afterwards the gel was washed 4 × 10 min each with 100 ml ddH_2_O and incubated with 100 ml sensitization solution (0.8 mM sodium thiosulfate) for 1 min. Following incubation*,* the gel was washed twice with ddH_2_O for 1 min each and stained for 30 min with 100 ml silver staining solution (12 mM silver nitrate) in the dark. Staining solution was rinsed off with ddH_2_O for 10 s and the gel transferred to a new glass dish containing 100 ml of a developing solution (100 mM boric acid 150 mM NaOH, 50 µM sodium thiosulfate 2% (w/v) glucose). Once sufficiently intense staining of the protein bands was observed, the developing solution was removed and the staining reaction was stopped by incubating for 30 min in 100 ml stop solution (330 mM Tris, 2% (v/v) acetic acid). In parallel, a streptavidin blot was performed with the same samples to detect enriched biotinylated proteins.

For protein identification, the gel was washed in ddH_2_O, the desired protein bands were excised and were subjected to LC–MS/MS analysis.

### LC–MS/MS analysis

#### In gel digest

Proteins were in-gel-digested using trypsin (Roche, Germany) according to Shevchenko et al.^61^. After digestion, the supernatant was collected in a new tube, dried down in a vacuum centrifuge and stored at − 20 °C. Dried samples were dissolved in 0.1% trifluoracetic acid for nano-LC–MS/MS analysis.

#### On-bead digest

Proteins bound on the streptavidin beads were washed three times with PBS and digested for 18 h at 37 °C and 800 rpm in an Eppendorf Thermomixer (Eppendorf, Germany) using 200 µg trypsin (Roche, Germany) per sample in 50 µl digest buffer (0.2% sodium deoxycholate, 10 mM Tris-(2-Carboxyethyl)phosphine hydrochloride, 40 mM chloracetamide, 100 mM ammonium bicarbonate). Samples were acidified with formic acid to a pH < 2 and centrifuged at 20,000×*g* for 10 min. Supernatants were loaded into a Stage Tip and peptides were purified according to Rappsilber et al.^62^. After Stage Tip purification, peptides were dried down in a vacuum centrifuge and stored at − 20 °C. Dried samples were dissolved in 0.1% TFA for nano-LC–MS/MS analysis.

#### Mass spectrometry analysis

Nano-LC–ESI–MS/MS experiments were performed on an Ultimate 3000 nano-RSLC (Thermo Fisher Scientific) coupled to an Exploris 480 mass spectrometer (Thermo Fisher Scientific) using a Nanospray-Flex ion source (Thermo Fisher Scientific). Tryptic peptides were directly injected to a pre-column (µ-pre-column C18 PepMap100, 300 µm, 100 Å, 5 µm × 5 mm, Thermo Fisher Scientific) and then separated on a NanoEase analytical column (NanoEase M/Z HSS C18 T3, 1.8 µm 100 Å 75 µm × 250 mm column, Waters GmbH, Germany) operated at constant temperature of 35 °C. Gradient elution was performed at a flow rate of 300 nl/min using a 30 min gradient with the following profile: 2–55% solvent B in 30 min, 55–95% solvent B in 10 min, isocratic at 95% solvent B for 5 min and 95–2% solvent B in 10 min followed by 10 min column re-equilibration at 2% solvent B. For the analysis of on-bead digested samples a longer gradient was used: 2–15% solvent B in 20 min, 15–30% solvent B in 27 min, 30–55% solvent B in 25 min, 55–96% solvent B in 10 min, isocratic at 96% solvent B for 10 min and 96–2% solvent B in 10 min followed by 10 min column re-equilibration at 2% solvent B. Solvents used were 0.1% formic acid (solvent A) and 0.1% formic acid in acetonitrile/H_2_O (80/20, v/v, solvent B).

The Exploris 480 and the Ultimate 3000 nano-RSLC were operated under the control of Xcalibur 4.4.16.14 and SII Xcalibur 1.6.0.6893 software. MS spectra (m/z = 200–2000) were detected in the Orbitrap at a resolution of 60.000 (m/z = 200) using a maximum injection time (MIT) of 50 ms and an automatic gain control (AGC) value of 3 × 10E6. Internal calibration of the Orbitrap analyzer was performed using lock-mass ions from ambient air as described in Olsen et al.^63^. Data dependent MS/MS mass spectra were generated for the 30 most abundant peptide precursors in the Orbitrap using high energy collision dissociation (HCD) fragmentation at a resolution of 15.000 (m/z = 200) and a normalized collision energy of 30. Further settings for MS/MS spectra included an isolation width of 1.6 Da, a MIT of 50 ms and an automatic gain control (AGC) value of 5 × 10E4. Internal calibration of the Orbitrap analyzer was performed using lock-mass ions from ambient air as described in Olsen et al.^63^.

#### MS data analysis

Mascot 2.6 (Matrix Science, UK) was used as search engine for protein identification. Spectra were searched against the *Drosophila melanogaster* reference proteome sequence downloaded in FASTA-format from UniProt^64^. Search parameters specified trypsin as cleaving enzyme, a 5 ppm mass tolerance for peptide precursors and 0.02 Da for fragment ions. Carbamidomethylation of cysteine residues was defined as fixed modification. Methionine oxidation, biotinylation of lysin and peptide N-termini as well as phosphorylation of serine and threonine were allowed as variable modification.

Mascot search results were imported into Scaffold version 4.10. (Proteome Software, USA). Peptide identifications were accepted with a peptide probability greater than 95.0% as specified by the Peptide Prophet algorithm^65^. Proteins had to be identified by at least five peptides and a protein probability of at least 99.9% to be accepted. Protein probabilities were assigned by the Protein Prophet algorithm^66^.

#### Protein quantification and data analysis for on-bead digest experiments

Raw files were imported into MaxQuant^67^ version 2.0.1.0 for protein identification and label-free quantification (LFQ) of proteins. Protein identification in MaxQuant was performed using the database search engine Andromeda. MS spectra and MS/MS spectra were searched against the drosophila melanogaster proteome sequence database downloaded from UniProt^64^. Reversed sequences as decoy database and common contaminant sequences were added automatically by MaxQuant. Mass tolerances of 4.5 ppm (parts per million) for MS spectra and 20 ppm for MS/MS spectra were used. Trypsin was specified as enzyme and two missed cleavages were allowed. Carbamidomethylation of cysteines was set as a fixed modification and protein N-terminal acetylation, methionine oxidation, serine/threonine/tyrosine phosphorylation and lysine biotinylation were allowed as variable modifications. The two latter modifications were not considered for quantification. The ‘match between runs’ feature of MaxQuant was enabled with a match time window of 1 min and an alignment time window of 20 min. Peptide false discovery rate (FDR) and protein FDR thresholds were set to 0.01. T-tests and Volcano plots were performed using Perseus version 1.6.14.0. Matches to contaminant (e.g., keratins, trypsin) and reverse databases identified by MaxQuant were excluded from further analysis. Proteins were considered for LFQ (label free quantification) if they were identified by at least two peptides. First, normalized LFQ values from MaxQuant were log2 transformed. Missing values were imputed by the “Replace missing values from normal distribution” function (Width = 0.3, Down shift = 1.8) implemented in Perseus drawing random numbers from a normal distribution representing low abundance intensity values. For Two-sided t-tests a FDR cut-off of 0.05 and an artificial within group variance (S0) of 0.1 was used. Volcano plots were used for pairwise comparison of sample groups. A p-value < 0.05 and a fold change > 2 were considered as significant change in protein abundance.

### Supplementary Information

Supplementary Information.

Supplementary Table 1.

Supplementary Table 2.

Supplementary Table 3.

## Data Availability

The LC–MS/MS datasets generated and analyzed during the current study have been uploaded as Excel sheets in the Supplementary Material.
