# Supplementary material for: In vivo identification of Drosophila rhodopsin interaction partners by biotin proximity labeling
Source: Sci Rep. 2024 Jan 23;14:1986. doi: 10.1038/s41598-024-52041-3 (PMC10805788; doi:10.1038/s41598-024-52041-3)

Suppl. Table 1S: Proteins identified in samples from excised gel slices

Excel File 1

Suppl. Table 2S: Proteins identified in samples from on-bead digestions

Proteins listed in Table 1 are highlighted in yellow.

Excel File 2

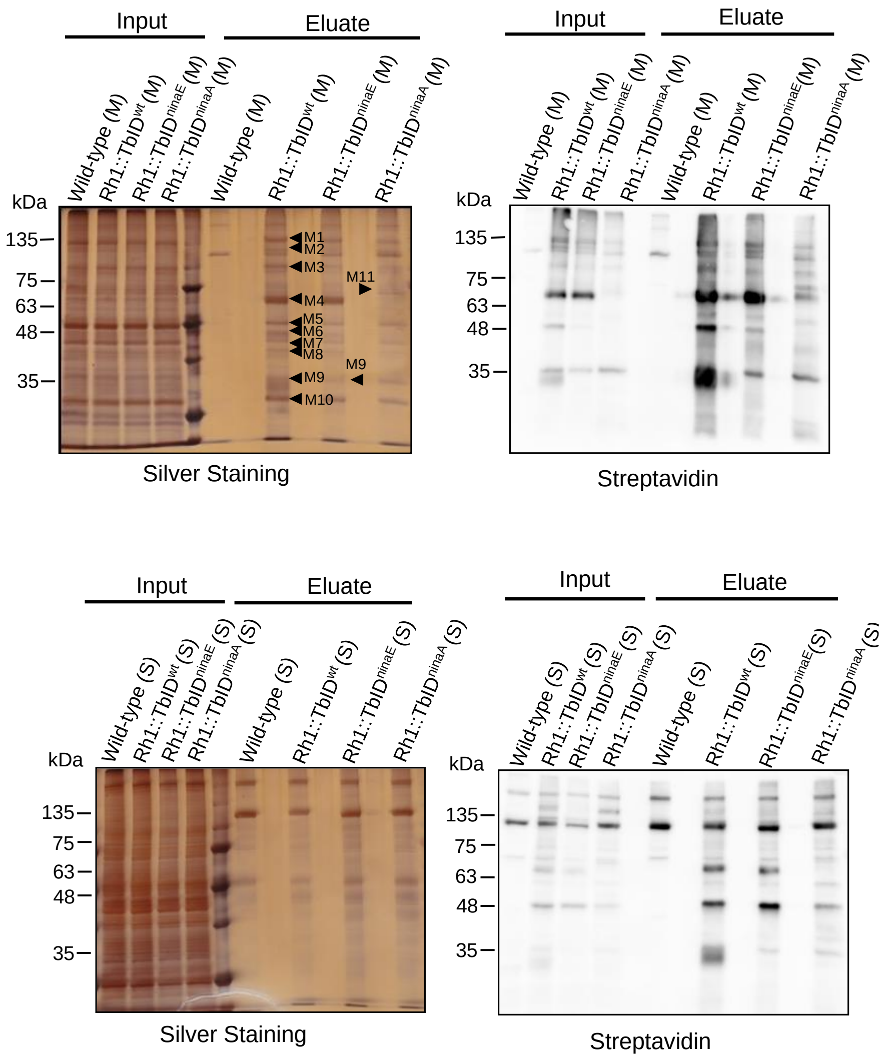

Suppl. Figure 1S: Streptavidin pulldown of biotinylated proteins for mass spectrometry analysis.

Wild-type flies and indicated mutants expressing Rh1::TbID were illuminated with white light for 12 h. Protein extracts of membrane proteins (M, upper panels) and of soluble proteins (S, lower panels) were incubated with streptavidin beads to pull down biotinylated proteins. Input that is extracted before streptavidin pulldown, and proteins eluted from the beads after the pulldown were loaded on 10 % SDS gels (equivalent of 1 and 25 heads per lane for input and eluate, respectively). One gel was stained with silver nitrate (left panels) and an equivalent gel was blotted and probed with streptavidin (right panels). Arrowheads indicate protein bands that were excised for LC-MS/MS analysis. The size of molecular weight markers in kDa is indicated on the left.

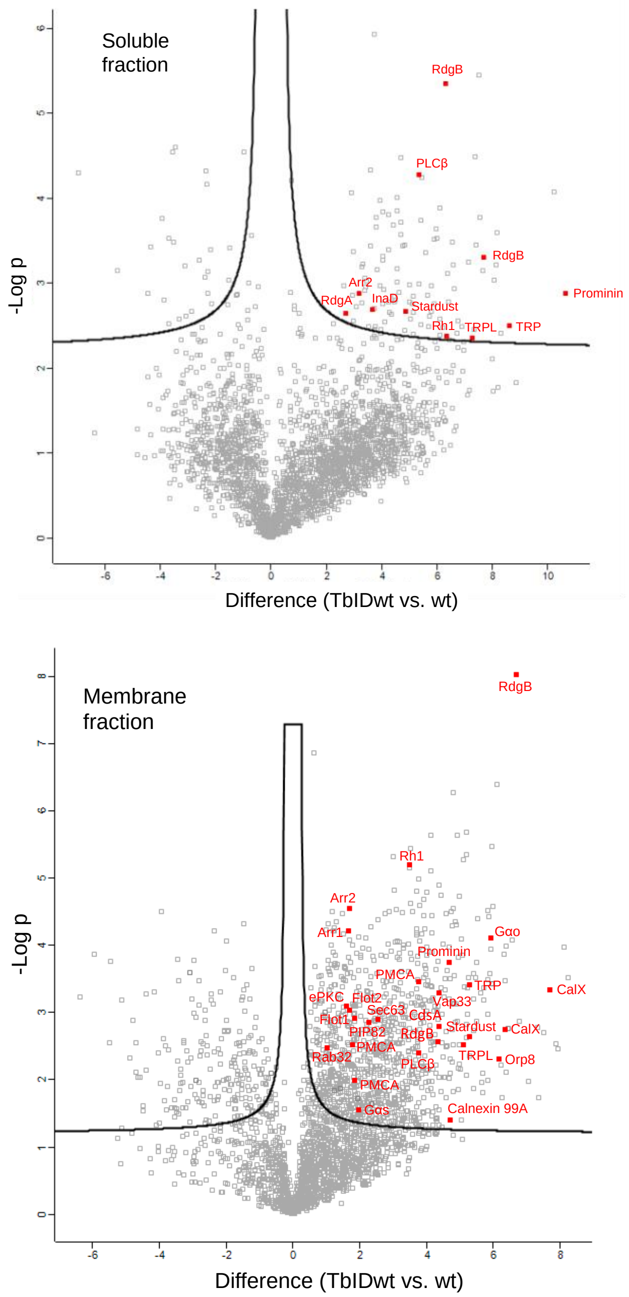

Suppl. Figure 2S: Volcano plots showing protein abundance differences between Rh1::TbIDwt and wt flies samples from on-bead digestion experiments.

Upper panel: soluble fraction, lower panel: membrane fraction. Proteins that were significantly increased or decreased in abundance according to the two-sided t-test (FDR 0.05, S0 = 0.1) are indicated by a solid black line. Proteins that were also identified in the in-gel digest experiment (Table 1) are highlighted in red.

Suppl. Figure 3S: MS/MS spectra of biotinylated peptides.

MS/MS spectra of biotinylated peptides from TRP (A-G), Rh1 (H,I), Prominin (J), NorpA (K), CdsA (L), Orp8 (M), DnaJ (N), and Use1 (O) are shown.

(A) MS/MS spectrum of peptide GSNTESDAEK.

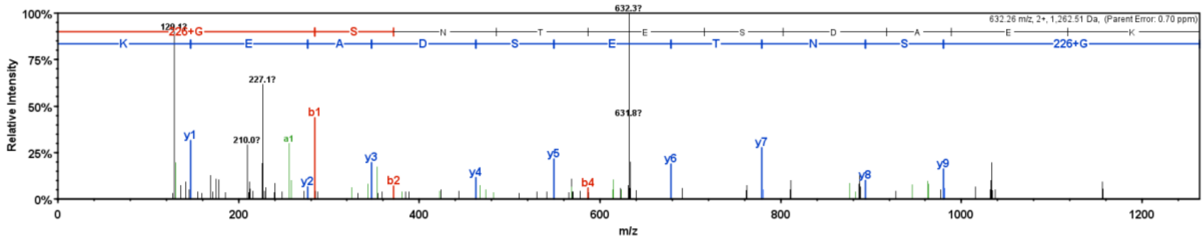

(B) MS/MS spectrum of peptide GDKDWNAIAR.

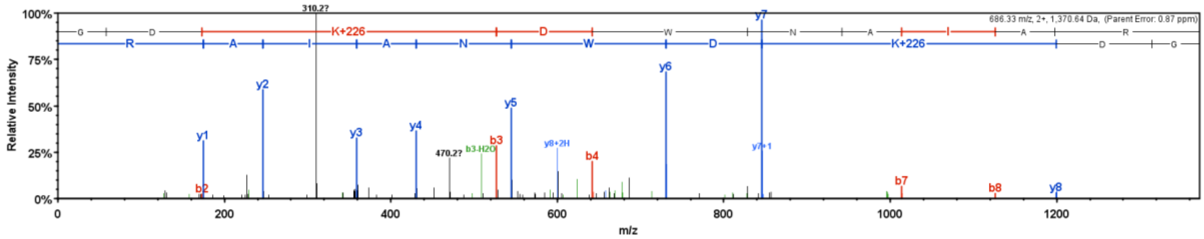

(C) MS/MS spectrum of peptide KNTFASDPIGSK.

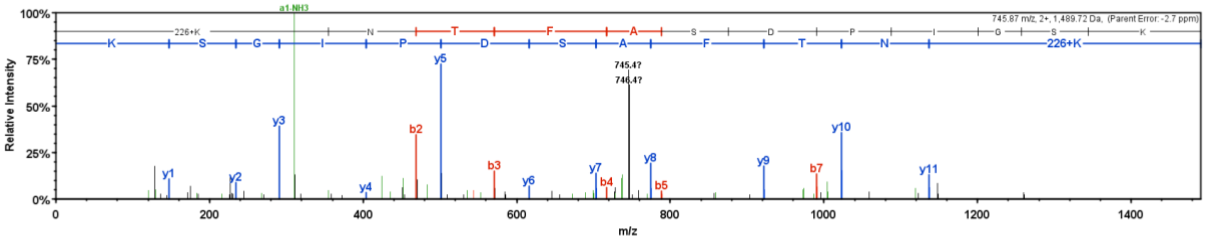

(D) MS/MS spectrum of peptide NTFASDPIGSKR.

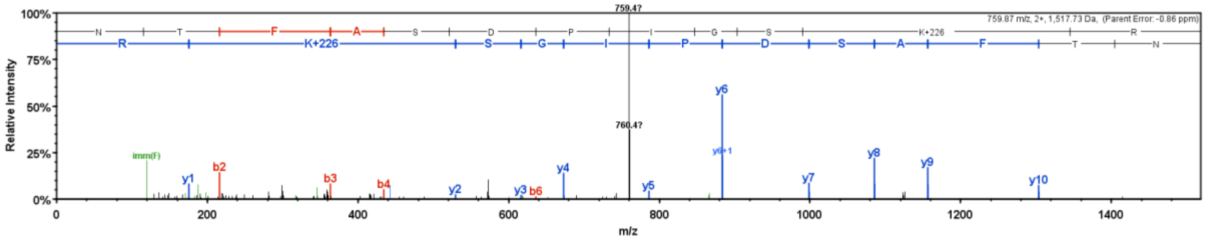

(E) MS/MS spectrum of peptide KPLDASGSKK.

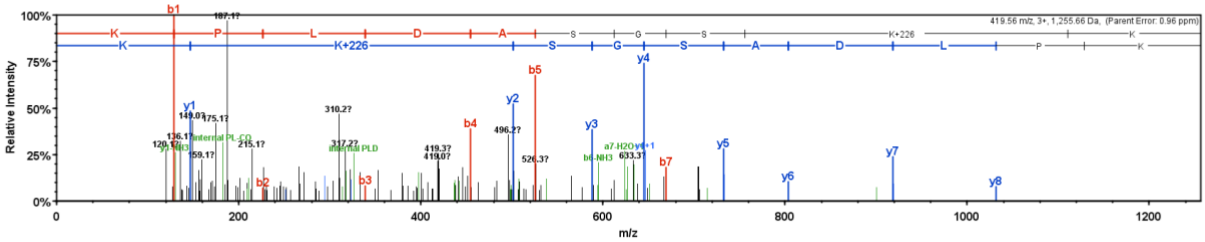

(F) MS/MS spectrum of peptide ASVKNVDEK.

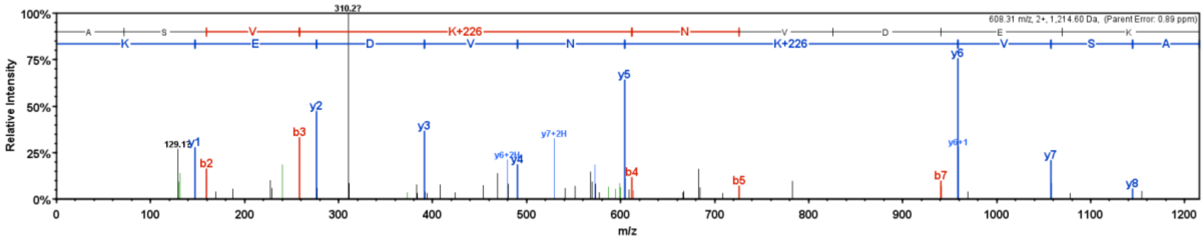

(G) MS/MS spectrum of peptide GKSTVIGR.

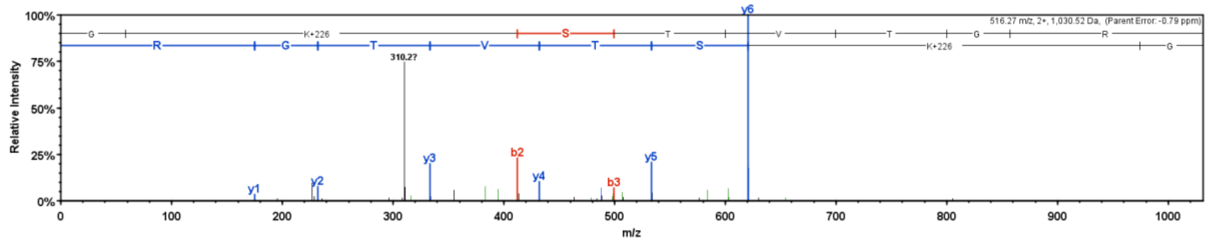

(H) MS/MS spectrum of peptide KMNVKSLR.

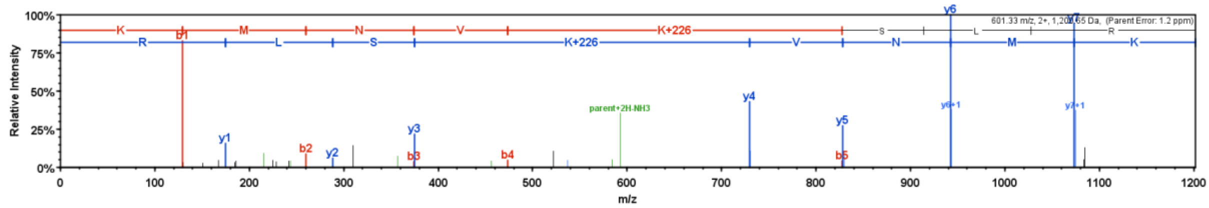

(I) MS/MS spectrum of peptide SSDAQSQATASEAESKA.

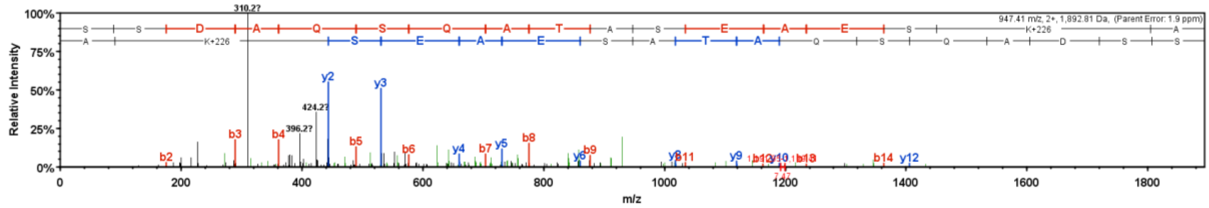

(J) MS/MS spectrum of peptide KILNIWQSR

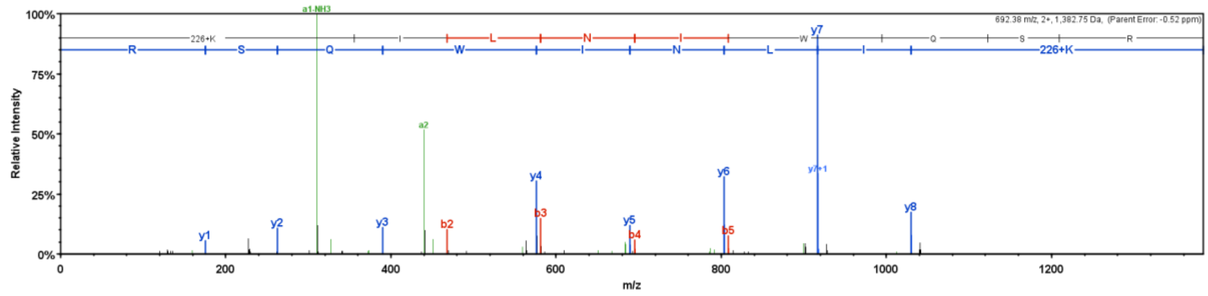

(K) MS/MS spectrum of peptide GFAGAAKQQNEQMK

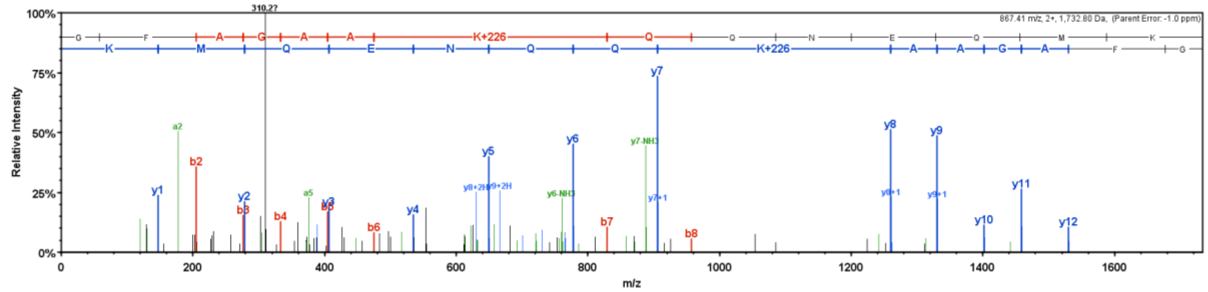

(L) MS/MS spectrum of peptide FVDELAKNLPQGTDK

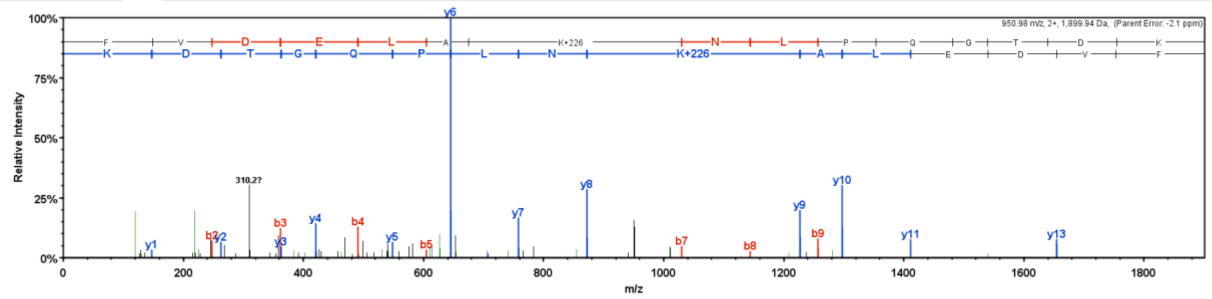

(M) MS/MS spectrum of peptide NKSLVLAPR

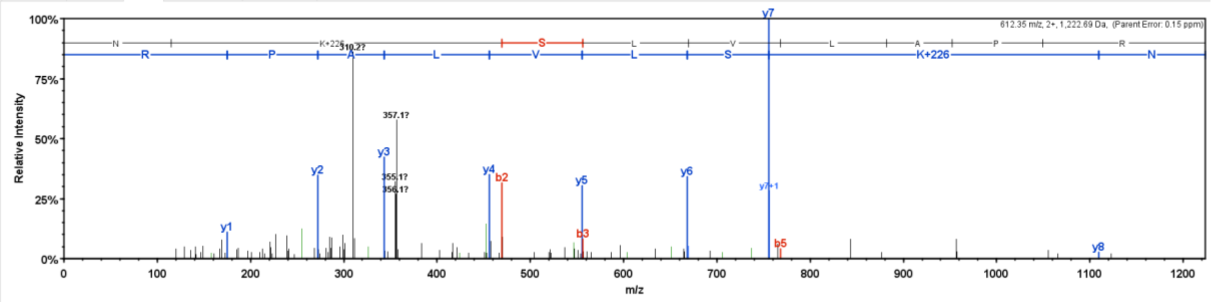

(N) MS/MS spectrum of peptide LSLSKNR

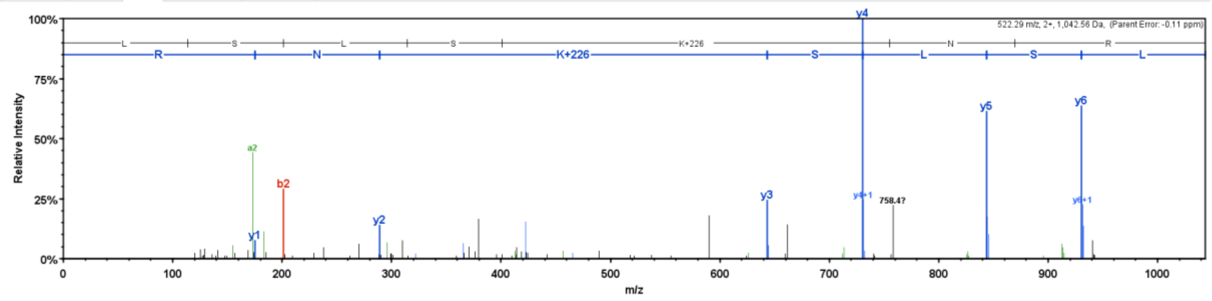

(O) MS/MS spectrum of peptide NLKEQTETANR

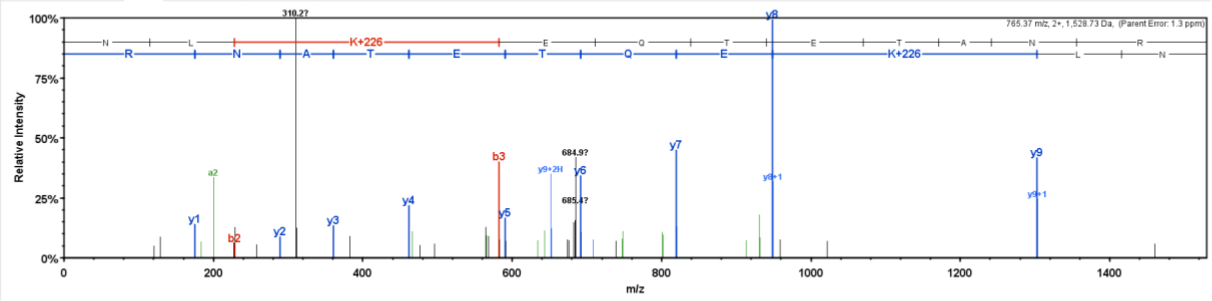

Suppl. Figure 4S: Full-length blots corresponding to cropped blots shown in figures as indicated.

Fig. 1B, upper panel

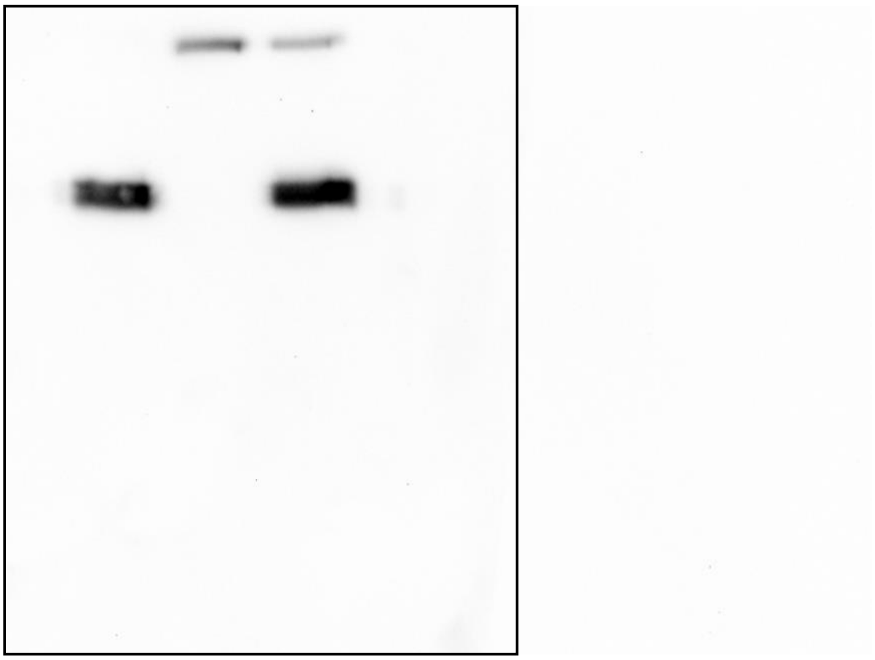

Fig. 1B, upper panel, post-contrast

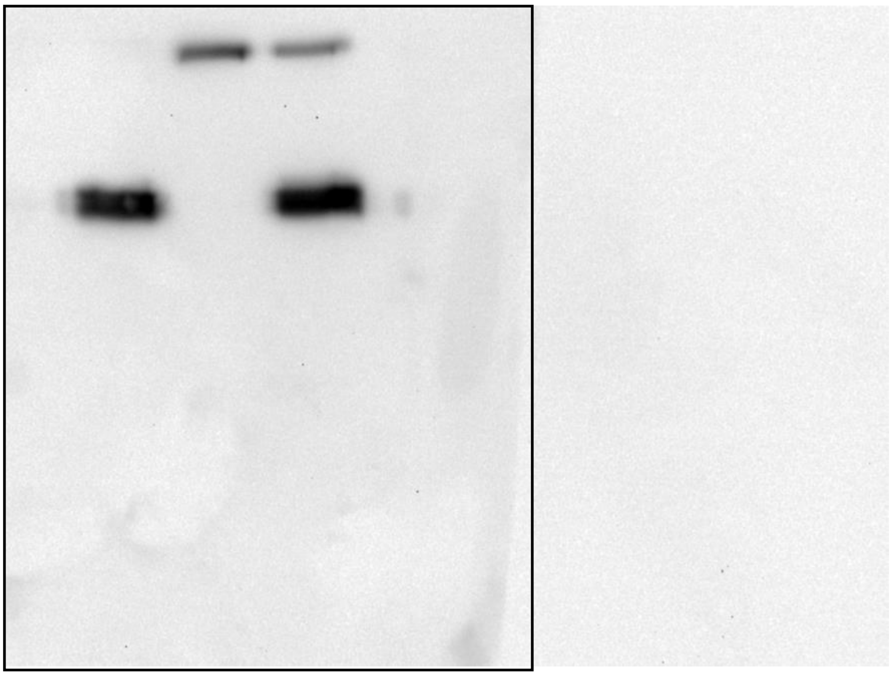

Fig. 1B, lower panel

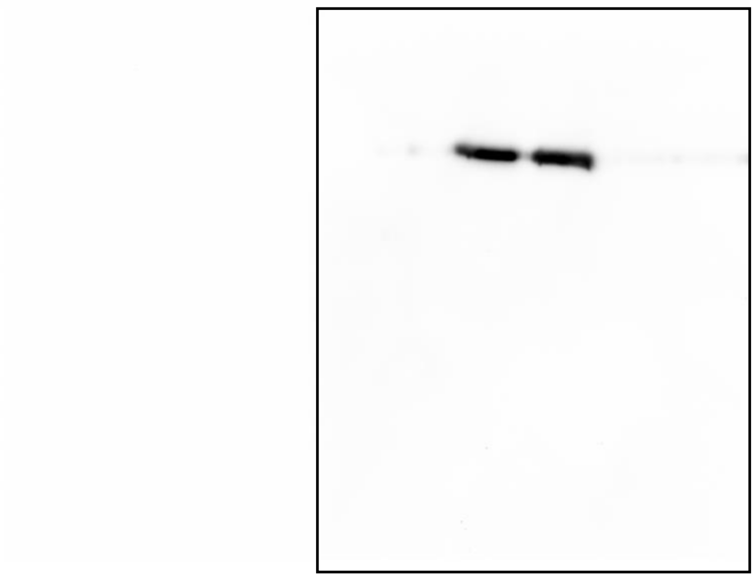

Fig. 1B, lower panel, post-contrast

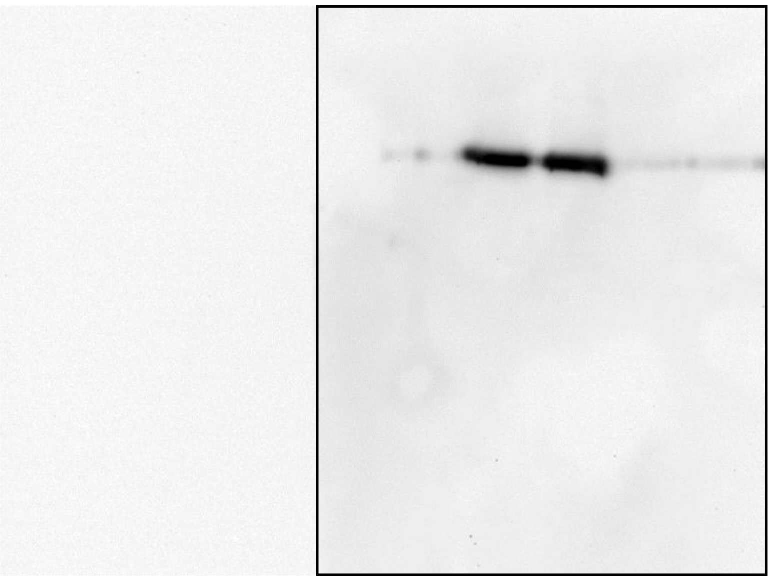

Fig. 1D, first recording

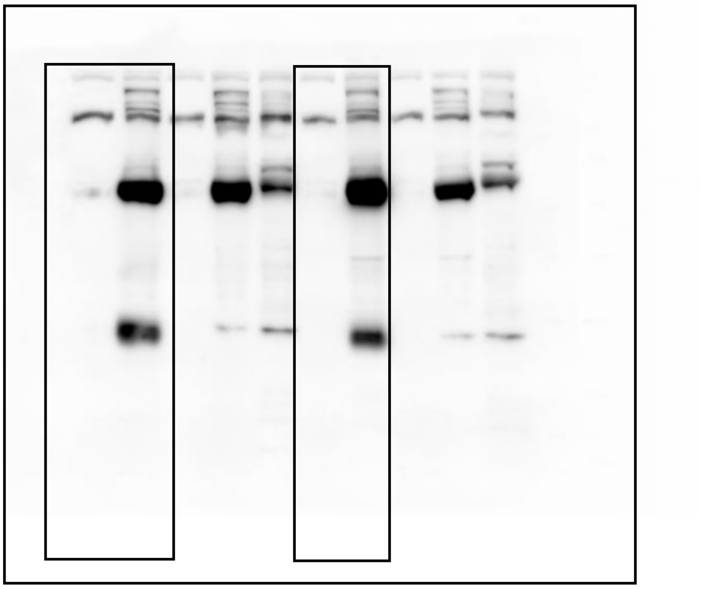

Fig. 1D, second recording

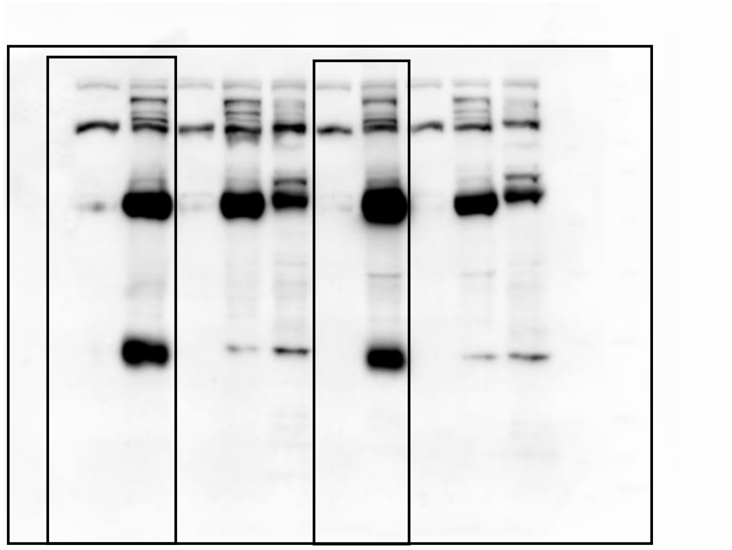

Fig. 3A, upper panel, first recording

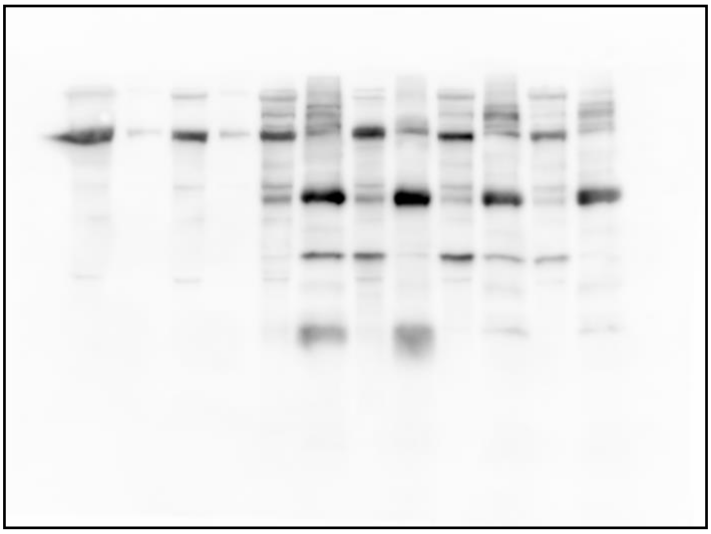

Fig. 3A, upper panel, second recording

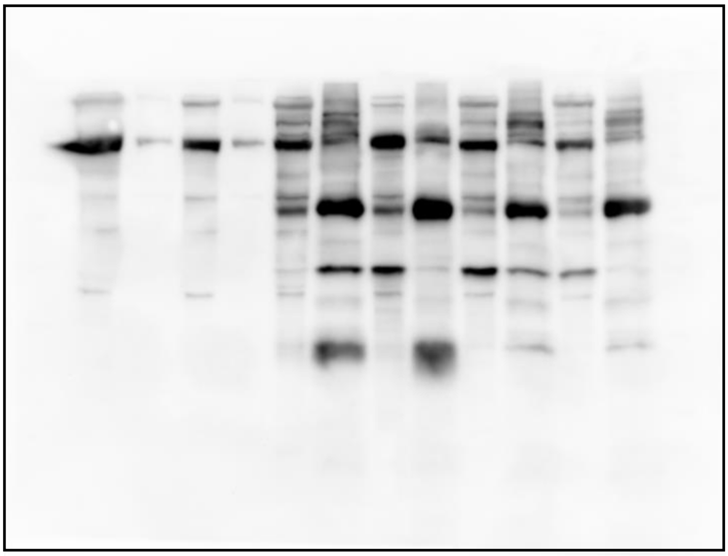

Fig. 3A, lower panel

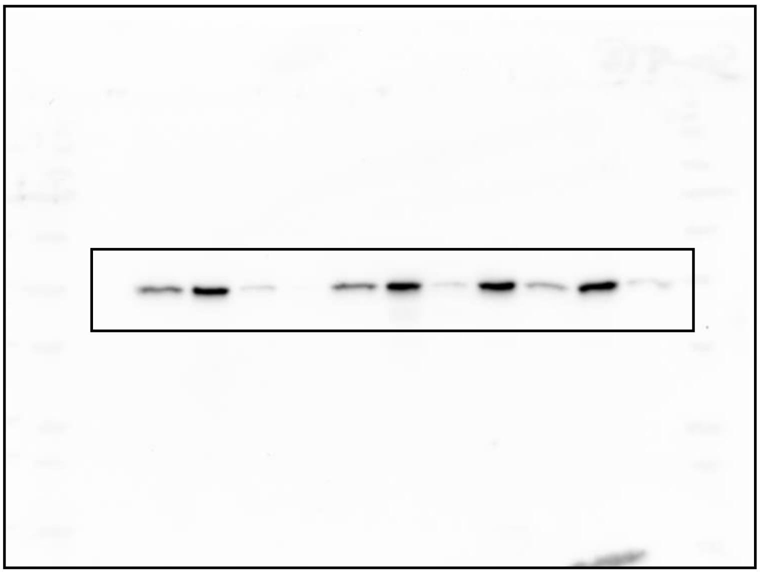

Fig. 3B, upper panel, first recording

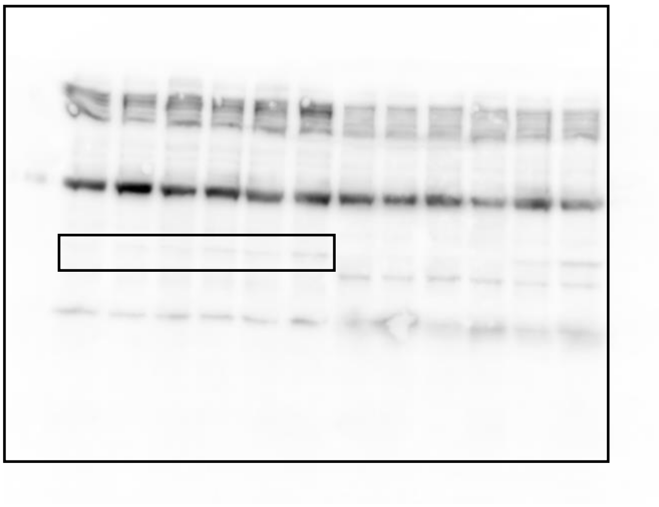

Fig. 3B, upper panel, second recording

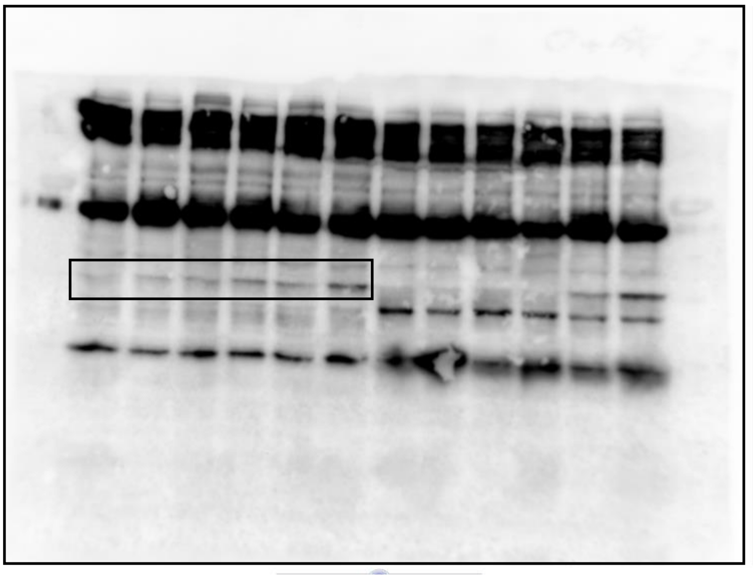

Fig. 3B, lower panel

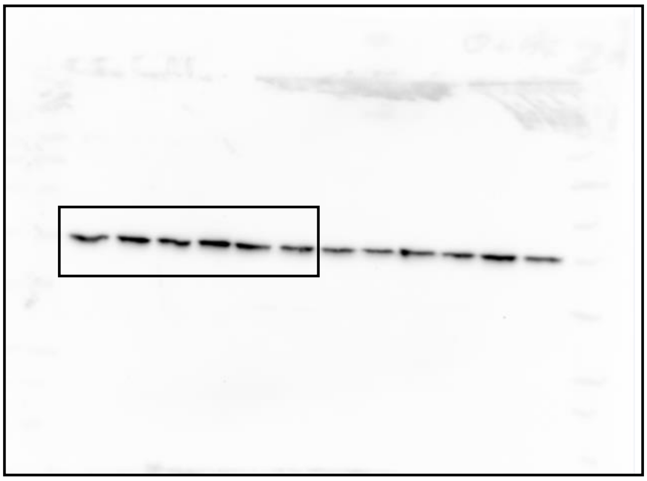

Fig. 4, first panel, first recording

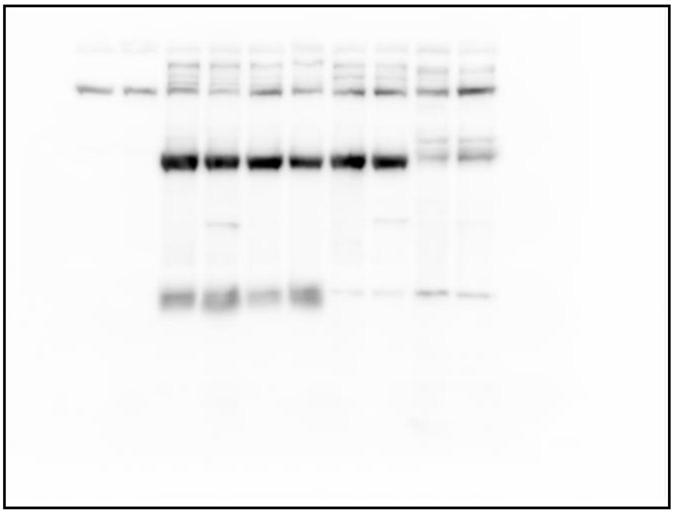

Fig. 4, first panel, second recording

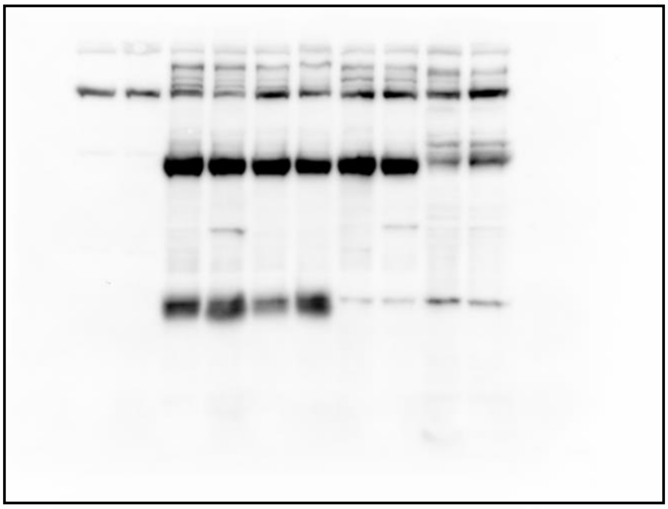

Fig. 4, second panel

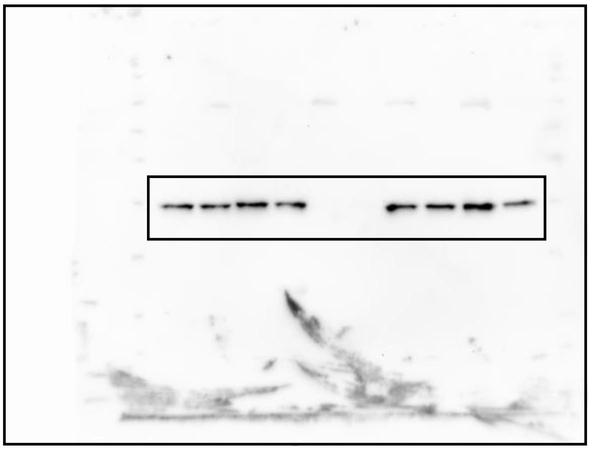

Fig. 4, third panel

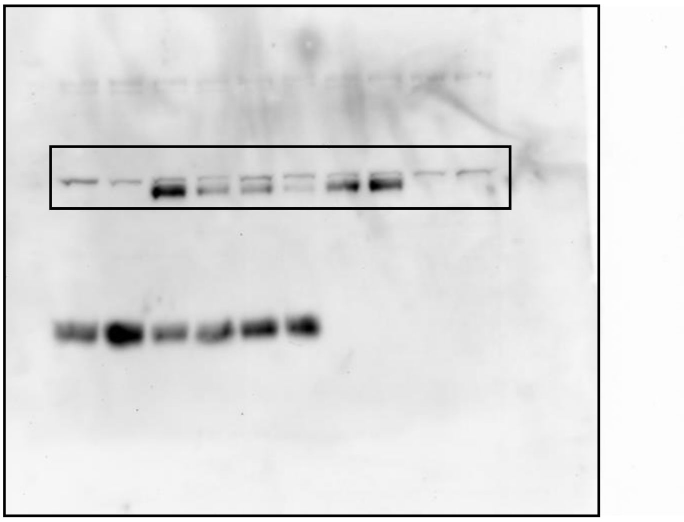

Fig. 4, fourth panel

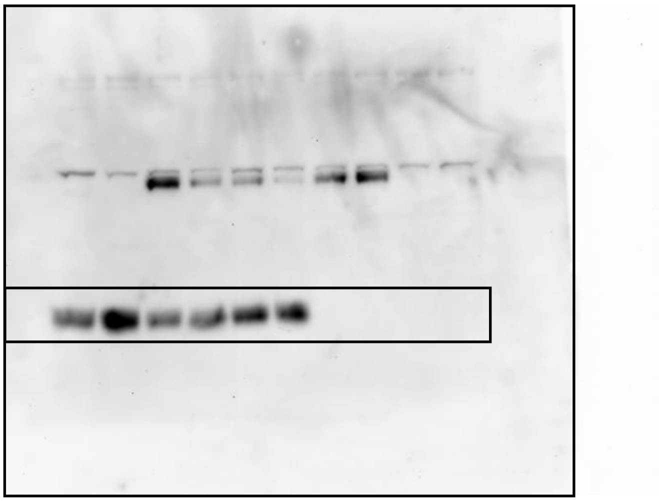

Fig. 5, right

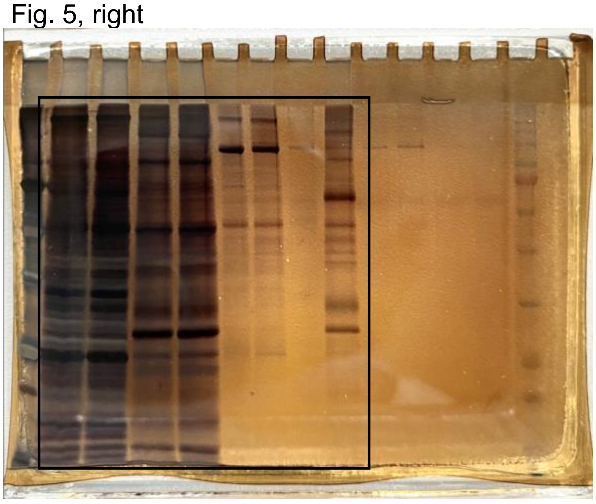

Fig. 5, left

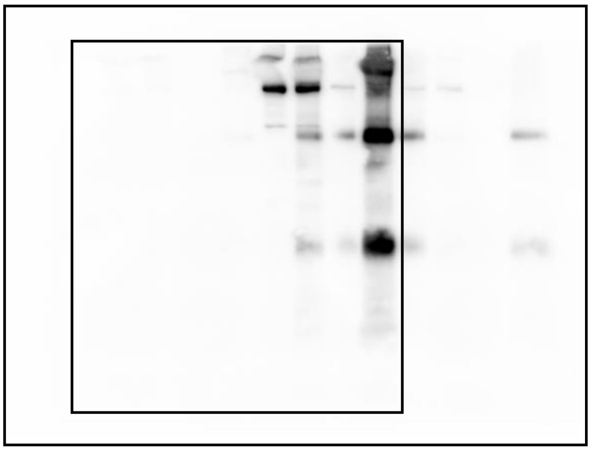

Fig. 5, left, second recording

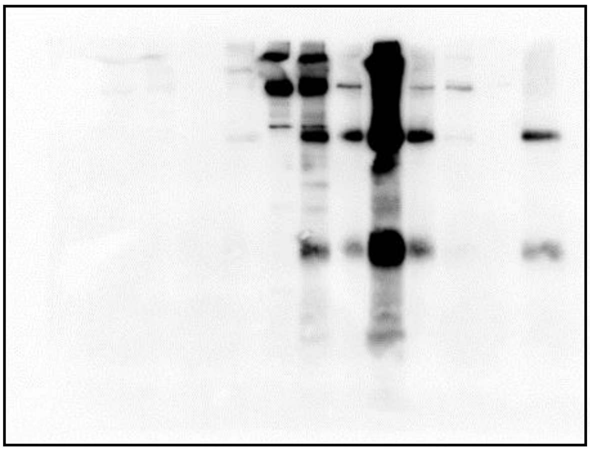

Fig. 6A, upper panel

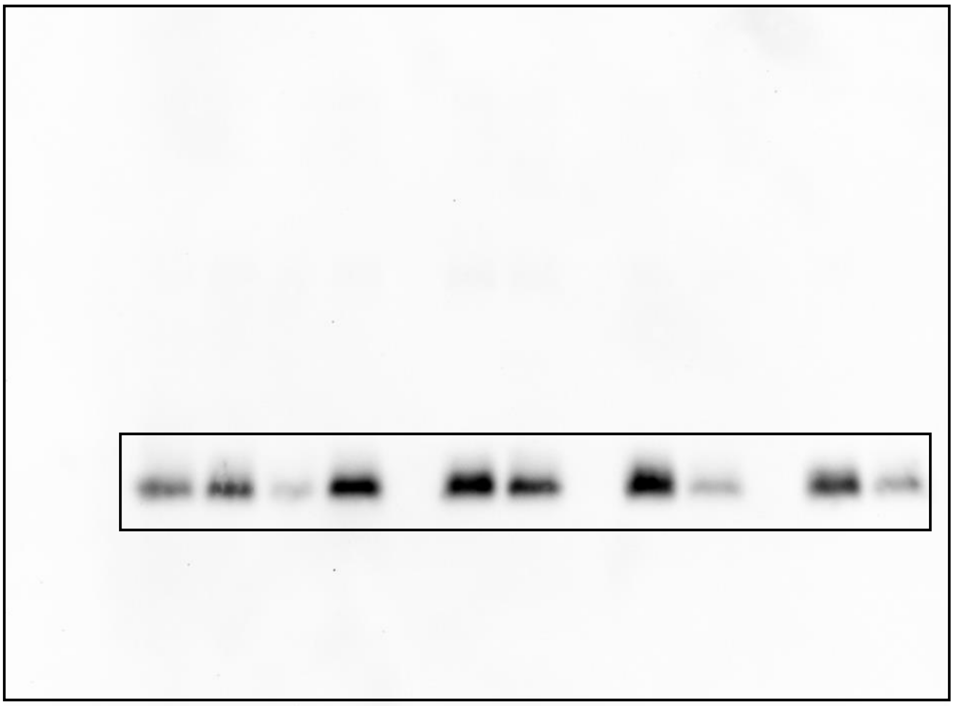

Fig. 6A, lower panel

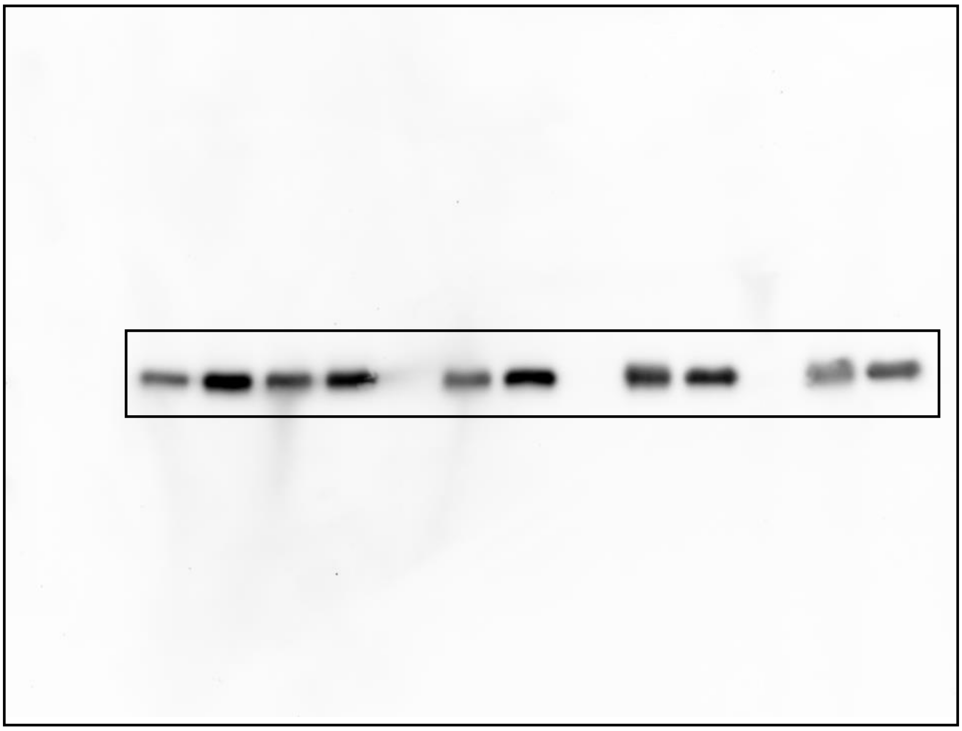

Suppl. Figure 1S, upper panel, right

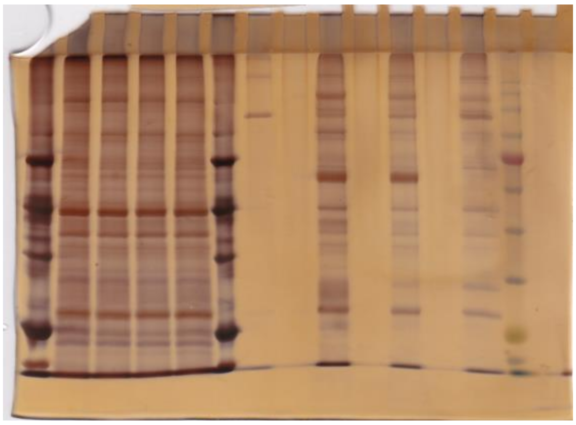

Suppl. Figure 1S, upper panel, left, first recording

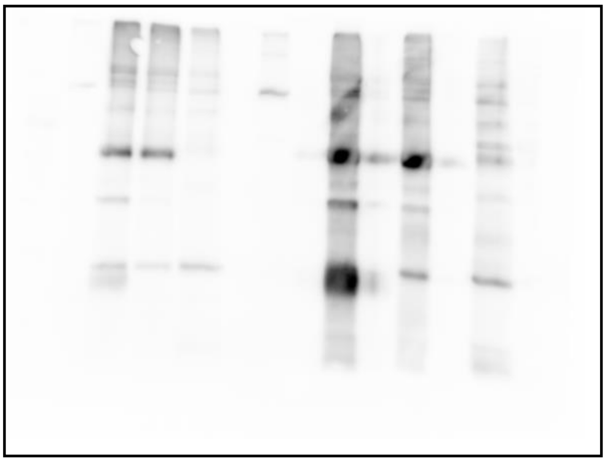

Suppl. Figure 1S, upper panel, left, second recording

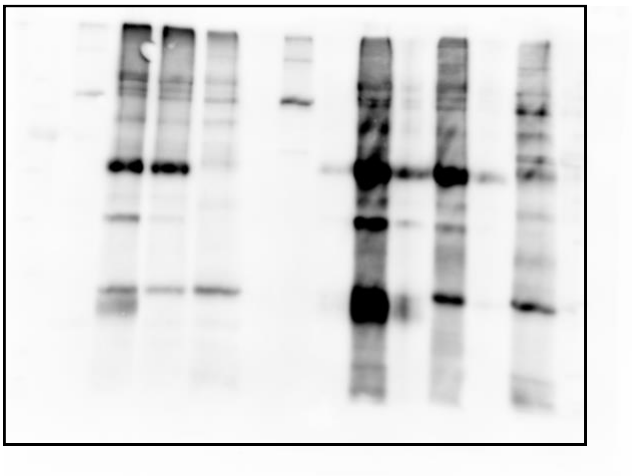

Suppl. Figure 1S, lower panel, right

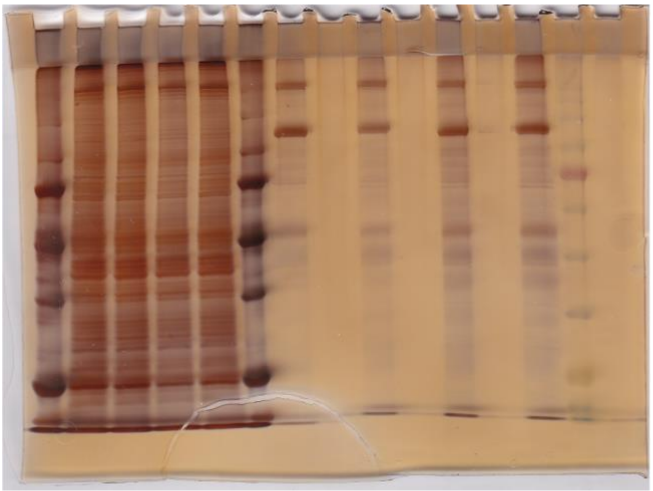

Suppl. Figure 1S, lower panel, left, first recording

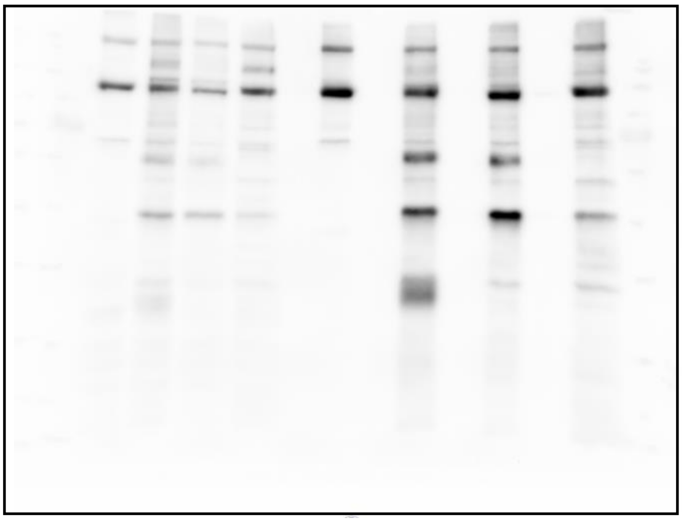

Suppl. Figure 1S, lower panel, left, second recording

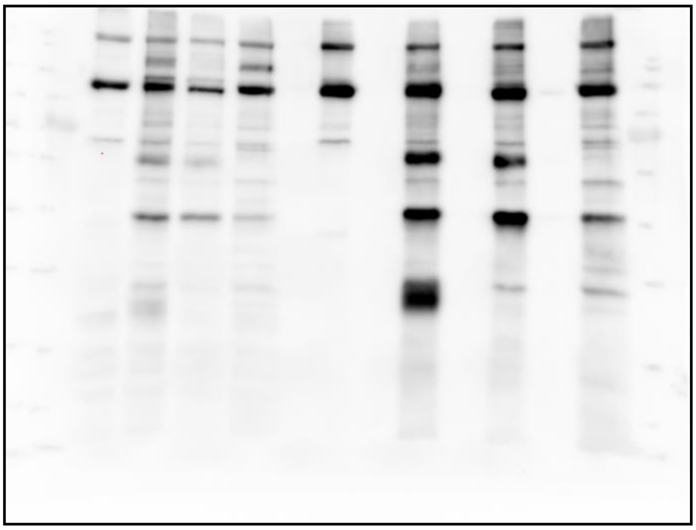

Supplement: Supplementary file 1 — Supplementary Information. [file 41598_2024_52041_MOESM1_ESM.pdf]
